# Intrahepatic CXCL10 is strongly associated with liver fibrosis in HIV-Hepatitis B co-infection

**DOI:** 10.1371/journal.ppat.1008744

**Published:** 2020-09-08

**Authors:** Kasha P. Singh, Jennifer M. Zerbato, Wei Zhao, Sabine Braat, Claire Deleage, G. Surekha Tennakoon, Hugh Mason, Ashanti Dantanarayana, Ajantha Rhodes, Jake W. Rhodes, Joe Torresi, Andrew N. Harman, Peter A. Revill, Megan Crane, Jacob D. Estes, Anchalee Avihingsanon, Sharon R. Lewin, Jennifer Audsley

**Affiliations:** 1 The Peter Doherty Institute for Infection and Immunity, The University of Melbourne and Royal Melbourne Hospital, Melbourne, Victoria, Australia; 2 Victorian Infectious Diseases Service, Royal Melbourne Hospital at the Peter Doherty Institute for Infection and Immunity, Melbourne, Victoria, Australia; 3 Department of Infectious Diseases, Alfred Health and Monash University, Melbourne, Victoria, Australia; 4 Centre for Epidemiology and Biostatistics, Melbourne School of Population and Global Health, University of Melbourne, Melbourne, Victoria, Australia; 5 Leidos Biomedical Research, Frederick National Laboratory for Cancer Research, Frederick, Maryland, United States of America; 6 Victorian Infectious Diseases Reference Laboratory, Royal Melbourne Hospital at the Peter Doherty Institute for Infection and Immunity, Melbourne, Victoria, Australia; 7 Centre for Virus Research, The Westmead Institute for Medical Research, Sydney, NSW, Australia; 8 The University of Sydney, Westmead Clinical School, Faculty of Medicine and Health, Sydney, NSW, Australia; 9 Department of Microbiology and Immunology, University of Melbourne at the Peter Doherty Institute for Infection and Immunity, Parkville, Victoria, Australia; 10 The University of Sydney, School of Medical Sciences, Faculty of Medicine and Health, Sydney, NSW, Australia; 11 Thai Red Cross AIDS Research Centre and Faculty of Medicine, Chulalongkorn University, Bangkok, Thailand; Vaccine Research Center, UNITED STATES

## Abstract

In HIV-hepatitis B virus (HBV) co-infection, adverse liver outcomes including liver fibrosis occur at higher frequency than in HBV-mono-infection, even following antiretroviral therapy (ART) that suppresses both HIV and HBV replication. To determine whether liver disease was associated with intrahepatic or circulating markers of inflammation or burden of HIV or HBV, liver biopsies and blood were collected from HIV-HBV co-infected individuals (n = 39) living in Bangkok, Thailand and naïve to ART. Transient elastography (TE) was performed. Intrahepatic and circulating markers of inflammation and microbial translocation were quantified by ELISA and bead arrays and HIV and HBV infection quantified by PCR. Liver fibrosis (measured by both transient elastography and liver biopsy) was statistically significantly associated with intrahepatic mRNA for CXCL10 and CXCR3 using linear and logistic regression analyses adjusted for CD4 T-cell count. There was no evidence of a relationship between liver fibrosis and circulating HBV DNA, qHBsAg, plasma HIV RNA or circulating cell-associated HIV RNA or DNA. Using immunohistochemistry of liver biopsies from this cohort, intrahepatic CXCL10 was detected in hepatocytes associated with inflammatory liver infiltrates in the portal tracts. In an in vitro model, we infected an HBV-infected hepatocyte cell line with HIV, followed by interferon-γ stimulation. HBV-infected cells lines produced significantly more CXCL10 than uninfected cells lines and this significantly increased in the presence of an increasing multiplicity of HIV infection. Conclusion: Enhanced production of CXCL10 following co-infection of hepatocytes with both HIV and HBV may contribute to accelerated liver disease in the setting of HIV-HBV co-infection.

## Author summary

Chronic hepatitis B infection is common in people living with HIV due to shared routes of transmission. Liver disease including fibrosis and cirrhosis is accelerated in those with HIV-hepatitis B coinfection, and although outcomes are improved with antiviral treatment, increased morbidity and mortality are still seen for unknown reasons.

We recruited people living with HIV-hepatitis B coinfection who had not been treated and assessed liver fibrosis using non-invasive means (transient elastography or ‘Fibroscan’) and liver biopsy. We measured multiple markers of HIV and HBV replication and inflammation in the liver as well as circulating in the blood to assess their relationship to liver fibrosis. We found that the inflammatory cytokine CXCL10 and its receptor CXCR3 in the liver were strongly associated with fibrosis. CXCL10 was localised to hepatocytes on fluorescent microscopy. We then infected a hepatocyte cell line with HIV and HBV and found that production of CXCL10 was greatly increased in the presence of both viruses. We conclude that increased CXCL10 in hepatocytes, as a result of co-infection of hepatocytes with HIV and HBV, may be an important new target for treatments to reduce liver disease in people living with HIV and HBV and should be further investigated.

## Introduction

In HIV-HBV co-infection, liver-related mortality is 17 times higher than HBV mono-infection [1]. Following the introduction of antiretroviral therapy (ART) that suppresses both HIV and HBV (HBV-active ART), liver-related mortality has significantly decreased [2, 3], however overall mortality, liver-related mortality and risk of hepatocellular carcinoma remain higher in treated HIV-HBV co-infection compared to treated HIV mono- or HBV mono-infection [4, 5]. Furthermore, liver disease progression continues to occur in ~10% of individuals on HBV-active ART [6]. Understanding the pathogenesis of liver disease progression in HIV-HBV co-infection is important for development of new strategies, supplementary to HBV-active ART, to minimise adverse clinical outcomes.

In untreated HIV infection, depletion of CD4+ T-cells from the gastrointestinal (GI) tract leads to increased microbial translocation and is associated with immune activation [7]. Studies have shown a relationship between microbial translocation, immune activation and liver cirrhosis in HIV-HCV co-infection [8], however in previous work, we did not find a relationship between plasma markers of microbial translocation and liver disease in HIV-HBV co-infection [9]. Therefore, factors other than microbial translocation may be important in driving liver disease in HIV-HBV co-infection.

In HBV infection, the pathogenesis of liver disease is multi-factorial. Studies have demonstrated that migration of activated T-cells to the liver are associated with liver flares and an increase in liver enzymes [10, 11]. Expression of interferon (IFN)-γ-inducible protein 10 (IP-10), also called C-X-C motif chemokine 10 (CXCL10), has been correlated with severity of injury in liver disease including viral hepatitis [12] (reviewed in [13]). CXCL10 binds to chemokine receptor 3 (CXCR3), which attracts CXCR3-expressing cells, including natural killer (NK) cells, activated T-cells and B-cells to sites of inflammation [14]. Immunohistochemistry and in vitro data suggest CXCL10 is secreted by hepatocytes in areas of inflammation [15, 16]. In chronic HBV, serum and intrahepatic CXCL10 concentrations increase during HBV flares [17] and correlate with HBV DNA, alanine aminotransferase (ALT) and progressive liver disease [18]; and elevated CXCL10 in blood is found in HIV mono-infection compared to controls [19]. We hypothesised that CXCL10 will also play an important role in liver fibrosis in HIV-HBV co-infection.

In addition to changes in chemokines, HIV may also accelerate fibrosis by direct effects on liver cells. In vitro and in vivo, HIV can infect hepatocytes [20–22], Kupffer cells [23], hepatic stellate cells [24] and T-cells recruited to the liver during inflammation [25]. HIV gp120 can bind to CC-chemokines expressed on hepatic stellate cells and trigger inflammation, migration [26] and pro-fibrotic gene expression in these cells [27]. Furthermore, HIV strains in the liver have distinct compartmentalised sequences when compared to brain and lymph nodes [28], so intrahepatic HIV DNA may represent a distinct reservoir. We therefore also sought to determine the relationship between intrahepatic HIV and liver fibrosis in HIV-HBV co-infection.

We performed a cross sectional study in people living with HIV and HBV and naïve to ART and analyzed paired liver biopsies and blood samples. Levels of intrahepatic mRNA for CXCL10 and CXCR3 correlated with liver fibrosis and immunohistochemistry demonstrated CXCL10 expression in peri-portal hepatocytes. Using an in vitro model, we demonstrated increased CXCL10 production from an HBV-infected hepatocyte cell line following HIV infection in the presence of interferon-γ. We propose a model where HIV infection of hepatocytes in the presence of HBV and local inflammation drives an increase in CXCL10, recruiting activated CXCR3+ cells to the liver; establishing a cycle of inflammation leading to liver fibrosis.

## Methods

### Study participants

People living with HIV-HBV co-infection (n = 39) were recruited from the HIV-Netherlands-Australia-Thai Research Collaboration (HIV-NAT), Thai Red Cross AIDS Research Centre, Bangkok, Thailand. Inclusion criteria were HIV antibody positive, HBV surface antigen (HBsAg) and/or HBV DNA positive on two occasions at least 6 months apart or HBsAg and/or HBV DNA positive with HBV core IgG antibody, 18 years or older and ART naïve. Exclusion criteria included detection of HCV antibody.

### Ethics statement

Written informed consent was obtained before enrolment and the study was approved by the Human Research Ethics Committees in Australia and Thailand.

### Liver fibrosis

Liver biopsies were scored by a single blinded pathologist according to the Metavir classification [29]. Liver stiffness was assessed by TE using Fibroscan (Echosens, Paris, France) by trained, experienced operators. We use TE cut-offs validated in HIV-HBV coinfection to stage fibrosis according to the Metavir system (F1-F4) such that F0/F1 < 5.9 kilopascals (kPa), F2 5.9 to < 7.6 kPa, F3 7.6 to < 9.4 kPa and F4 ≥ 9.4.kPa [30].

### Markers of microbial translocation, and immune mediators in plasma and cell culture

Lipopolysaccharide (LPS), CXCL10 and CCL2 were quantified as previously described [9]. Plasma soluble (s) CD14 and High mobility group box 1 (HMGB1), a marker of cell damage and death [31] were measured by ELISA in duplicate according to the manufacturer’s instructions (R&D Systems, McKinley Place, MN and IBL International GMBH, Hamburg, Germany respectively). For in vitro experiments, CXCL10 was quantified by single-analyte ELISA according to the manufacturer’s instructions (Human CXCL10/IP-10 ELISA MAX Deluxe Set, Biolegend, San Diego, CA).

### Plasma HBV DNA and HBV surface antigen (qHBsAg)

HBV DNA was quantified using the Abbott m2000sp/m2000rt Realtime HBV test (Abbott Laboratories, Abbott Park, Ill, USA) with a lower limit of quantification of 10 IU/ml. HBsAg was quantified as described [32].

### HIV in peripheral blood CD4+ T cells

HIV DNA was quantified in purified CD4+ T-cells as described previously [33] and normalized to cell number by quantification of the *CCR5* gene [33]. Cell associated (CA-)unspliced (US) HIV-RNA was quantified using a semi-nested real-time quantitative PCR assay with syber green included in the second round PCR and the result was normalised to cell number by quantification of 18S as previously described [33–35]. Primers were modified to recognise HIV clade A/E, as previously described [36], which is the dominant clade in Thailand (primers in S1 Table). The lower limit of detection for HIV RNA and HIV DNA was one copy per well. If there was no HIV PCR signal, this was recorded as zero and if there was a detectable signal but <1, this was recorded as 0.5 copies.

### HIV and HBV in liver biopsies

Liver biopsy samples were placed in RNA*later* Stabilization Solution (ThermoFisher Scientific Baltics UAB, Vilnius, Lithuania), and stored at -80°C until processing by scalpel disaggregation then lysis and homogenization using Qiagen RLT buffer and a Qiagen Qiashredder column (Qiagen, Hilden, Germany). HIV DNA and CA-US HIV RNA were then quantified as described for CD4+ T-cells.

Extracted DNA was also used to measure HBV covalently closed circular (ccc)DNA and relaxed circular (rc)DNA as previously described [37] with the following modifications: extracted liver DNA was treated with Exonuclease I and III (New England Biolabs, Ipswich, MA, USA) to eliminate replicative intermediates. To each liver DNA sample, 1 μL (20 units) Exo I & 0.25 μL (25 units) Exo III, in 1X NEBuffer 1, and incubated at 37°C for 3 hrs, followed by heat-treatment at 80°C for 20 min, then cccDNA molecular analysis performed. Lower limit of quantification was 0.002 copies/genome equivalent.

### Quantitative Real Time-PCR (qRT-PCR) for CXCL10, CXCR3 and IFN in liver biopsies

qRT-PCR for CXCL10, CXCR3 and IFN mRNA was performed as previously described [38]. (primers in S1 Table). Relative amounts of PCR product were determined using the comparative cycle threshold delta-delta Ct method, where the amount of target cDNA was normalized to the internal control, ribosomal protein large PO (RPLPO) for CXCL10/CXCR3 and to glyceraldehyde 3-phosphate dehydrogenase (GAPDH) for IFN, and expressed relative to the baseline unstimulated control (assigned a mean value of 1). PCR efficiencies of the housekeeping gene and target gene were similar.

### Immunohistochemistry and quantitation of CXCL10, LPS and myeloperoxidase (MPO10) in liver biopsies by quantitative image analysis

Immunohistochemical staining and quantitative image analysis were performed as previously described [39]. Briefly, slides were incubated with antibody to CXCL10 (Rabbit; 4:100, OriGene, Rockville, Maryland), LPS (Mouse; 1:100, Abcam, Cambridge UK) or myeloperoxidase (MPO) (Rabbit; 1:1000, DAKO (Agilent) Santa Clara, CA) then rabbit or mouse Polink- 2 horseradish peroxidase (HRP) and developed with Immpact DAB (3,3′-diaminobenzidine; Vector Laboratories, Burlingame, CA), or red-AP (Vector Laboratories) then counterstained and mounted in Permount (Thermo Fisher, Waltham, MA), and scanned at high magnification (x200) using the ScanScope CS System (Aperio Technologies, Vista, CA), yielding high-resolution data from the entire tissue section. Representative regions of interest (500 mm^2^) were identified and high-resolution images extracted from these whole-tissue scans. The percentage area positive for myeloperoxidase (MPO) (a neutrophil marker), LPS and CXCL10 was quantified using Photoshop CS5 and Fovea tools.

Liver biopsy samples from people without HIV, HBV and HCV infection who were undergoing liver biopsy for other indications, were used as controls for CXCL10 immunohistochemical staining.

### Fluorescence microscopy

To phenotype the cells expressing CXCL10, fluorescence microscopy was performed using an RNAscope approach with a probe targeting CXCL10 (#311851, ACD, Newark, CA), combined with immunofluorescence using nuclear stain (DAPI) with antibodies to either CD4 (Rabbit; 1:100, Abcam), CD8 (Rabbit; 1:100, Thermo Fisher), NKp44 (Mouse; 1:500, Biolegend, San Diego, CA) with NKp46 (Mouse; 1:500, R&D Systems, Minneapolis, MN) and CD57 (Mouse; 1:100 Invitrogen (Carlsbad, CA) for natural killer cells, CD68 (Mouse; 1:500, Biocare, Pacheco, CA) with CD163 (Mouse; 1:500, Novo Castra, Newcastle Upon Tyne, UK) for myeloid cells or Hepatocyte monoclonal antibody (MA5-12417-OCH1E5, Thermo Fisher) for hepatocytes. Up to four sections of 5μm each were screened and using a confocal microscope (Olympus, Fluoview FV10i), representative pictures of CXCL10+ cells were taken to evaluate the type of cells expressing CXCL10 in liver biopsy specimens.

### In vitro model of hepatocyte response to IFN-γ

We used the human hepatocellular carcinoma-derived hepatocyte cell line HepG2 (obtained from the American Type Culture Collection) and HepG2-derived AD38 cell line (kindly donated by Professor Stephen Locarnini, Melbourne, Australia) which is stably transfected with a complementary DNA (cDNA) of pregenomic (pg) RNA and mimics the in vivo HBV life-cycle including DNA and RNA replicative intermediates, HBsAg and HB core Ag production [40]. Cells were plated and treated with recombinant human IFN-γ (R&D systems, 500ng/ml), and the synthetic tripalmitoylated lipopeptide Pam3Cys-SerLys4 (P3CSK4) (InvivoGen, 1000ng/ml) for 24 hours at 37°C for all experiments. P3CSK4 mimics the acylated amino terminus of bacterial lipoprotein and is recognised by toll like receptors (TLR)-1/2. Based on previous experiments, we found that P3CSK4 induced CXCL10 production, which was not observed with TLR4 agonist LPS [9].

For protein stimulation experiments 1000U/mL Type 1 IFN (R&D systems, 11200–2), 100 IU/ml HBsAg, 100 IU/ml HBcAg (kindly donated by Professor Mala Maini, London, United Kingdom), 100ng/ml HIV gp120 (Bal strain, R5-tropic), 0.5nM or 5nM Lyovec ssHIV RNA or a scrambled RNA control (InvivoGen, San Diego, CA) were added with IFN-γ/P3CSK4.

For infection experiments with HIV, HepG2 cells were seeded +/- antiretroviral (ARV) drugs added to the media, and then infected with a single round vesicular stomatitis virus (VSV) glycoprotein G-pseudotyped NL4.3 virus with an envelope deletion expressing green fluorescent protein (VSV-G-NL4.3Δenv-eGFP) (kindly donated by Damian Purcell, University of Melbourne, Australia) [22] at multiplicity of infection (MOI) 0.5, 0.25, 0.125 or 0.0625 or a VSV-G-pseudotyped NL4.3 virus containing a luciferase reporter without an envelope and Vpr (VSV-G-NL4.3-Luc-Δenv-Δvpr) (NIH AIDS reagent program) at MOI 1, 0.5, 0.25, 0.125. The ARV drugs used were: raltegravir (RAL) 1μM, efavirenz (EFV) 300 nM and T20 2μg/mL. These agents are active against HIV but not against HBV.

After 16 hrs, cells were washed with PBS and then stimulated with IFN-γ/P3CSK4 and incubated for a further 24 hours. Supernatants were collected and mixed with 0.3% NP-40 and stored at -80°C for subsequent ELISA. Cells were stained for viability using amine reactive dye (LIVE/DEAD Fixable Near-IR Dead Cell Stain Kit, ThermoFisher Scientific), and analysed using flow cytometry for detection of viability and eGFP expression on a BD LSR2 Fortessa flow cytometer.

### Statistical analysis

Variables were summarized using median (25^th^-75^th^ percentiles) or number (percentage) for continuous and categorical data, respectively. Spearman’s rank correlations were calculated between liver-related variables and cytokines, immunological and viral parameters and presented in a heatmap. For a sample size of 39, the relationship between p-values and Spearman’s rank correlation was p<0.05 if r^2^>0.30; p<0.01 if r^2^ > 0.43, and p<0.001 if r^2^> 0.60. The correlation between fibrosis grade by TE and liver biopsy was described using Goodman-Kruskal gamma.

We performed regression analyses adjusted for CD4+ T cell count to estimate the association between plasma and liver immune markers, cytokines and virological parameters against liver related outcomes. Fibrosis by TE (kPa), CXCL10, CXCR3, IFN-γ and LPS were log_e_ transformed due to skewness. Continuous liver related outcomes were analysed using linear regression. For log_e_ transformed outcome variables, exponentiated regression coefficients have also been included. For the regression coefficient (β), one unit increase in the covariate results in (exp[β]-1) x 100 percentage change in the outcome.

Metavir scores were analysed using ordered logistic regression. Partial eta squared values were obtained for the linear regression models to quantify the effect size. Using Cohen’s benchmarks [41] to categorise partial eta-squared (η2) as small (η2 = 0.01), medium (η2 = 0.06), and large (η2 = 0.14), the size of the estimated association between exposure and outcome was quantified. For an interpretation of the biological importance or clinical relevance of the results, the two-sided 95% confidence interval was considered as well as the magnitude of the partial eta-squared for linear regression and the odds ratio for logistic regression.

Clinical and laboratory parameters were compared between groups defined by detectable/undetectable HIV in liver, using the Wilcoxon Rank-Sum test.

Effect sizes (fold-change/geometric mean ratio) between different in vitro conditions for both cell lines were estimated using analysis of variance of log_10_ transformed data with all data points treated as independent. *P*-values were obtained using the Wilcoxon Rank-Sum test for each pair of stimuli within each cell line, based on the average of the (log_10_) replicates for each stimulus within each experiment.

A two-sided 5% level of significance was used. No adjustment for multiple testing was undertaken. Based on our previous work and findings from HIV-HCV, we hypothesized that CXCL10 will play an important role in liver fibrosis in HIV-HBV co-infection. Heatmaps were created using GraphPad Prism version 7.0b for Windows, GraphPad Software, La Jolla California USA, www.graphpad.com, and analyses were conducted using STATA/SE Version 15.1 for Windows.

## Results

### Study participants & clinical features

Participants were young, predominantly male, HBeAg positive, with a median (25^th^– 75^th^ percentiles) CD4+ T-cell count of 360 (221–462) cells/μl (Table 1). Immunological and virological parameters measured in plasma and liver are summarised in Table 1. In most participants, liver fibrosis was mild (<F3). TE (grade) results showed a significant positive association with liver biopsy grade (Gamma 0.54, p = 0.003)[42].

**Table 1:** Demographic and clinical characteristics (n = 39).

**Clinical**	
Age, years	31.9 (25.3–35.8)
Sex male, % (n)	89.7 (35)
Alcohol intake, % (n)	
Never	43.6 (17)
Ever	56.4 (22)*
**Laboratory**	
CD4, cells/μL	360 (221–462)
CD8 total, cells/μL	965 (662–1334)
HIV RNA, log_10_copies/mL	4.9 (4.5–5.5)
HBV DNA, log_10_IU/mL	7.4 (2.6–8.1)
HBeAg positive, % (n)	64.1 (25)
Alanine Aminotransferase (ALT), U/L	38 (27–62)
Aspartate Aminotransaminase (AST), U/L	33 (26–46)
Alkaline phosphatase (ALP), U/L	70 (61–78)
γ-glutamyl transferase (GGT), U/L	32 (19–61)
**Fibrosis assessment**	
Transient elastography (TE), kPa TE, kPa	6.3 (5.3–8.3) 6.3 (5.3–8.3)
TE, Fibrosis grade, % (n)	
F0-F1	46.2 (18)
F2	35.9 (14)
F3	10.3 (4)
F4	7.7 (3)
Liver biopsy fibrosis grade (Metavir), % (n)	
F0	61.5 (24)
F1	30.8 (12)
F2	0.0 (0)
F3	7.7 (3)
F4	0 (0)

All values are presented as median (25th-75th percentiles) unless otherwise stated.

*Includes those indicating drinking less than monthly (n = 8) monthly (n = 9), weekly (n = 4) or daily (n = 1)

### Associations with liver fibrosis as measured by both TE and biopsy

**Fig 1:**
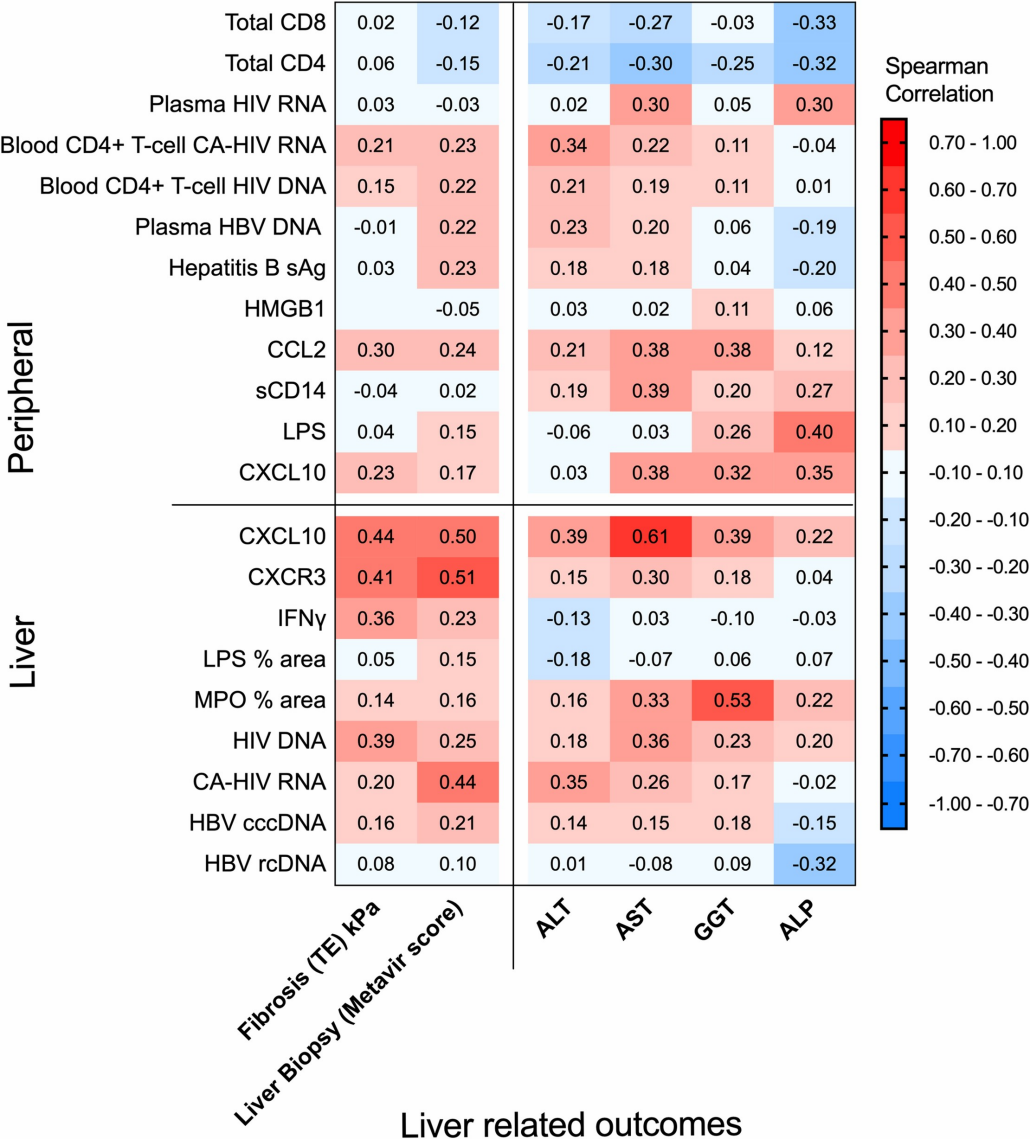
Correlations between HIV, Hepatitis B and inflammatory markers in the liver and blood. Heatmap showing the significant associations by Spearman correlation between intrahepatic CXCL10, CXCR3 and HIV with liver related outcomes including fibrosis and liver enzymes (left hand panel) and between the related intrahepatic markers CXCL10, CXCR3 and IFN-γ and liver and HIV in the liver (right hand panel). P-value < 0.05 if absolute value of Spearman’s correlation is at least 0.30, < 0.01 if at least 0.44 (n = 39). HIV human immunodeficiency virus, HBV hepatitis B virus, CA cell associated, sAg surface antigen, ALP alkaline phosphatase; GGT γ-glutamyl transferase, ALT Alanine transaminase, AST Aspartate transaminase, HMGB1 high mobility group box-1, CCL-2 C-C motif chemokine 2, sCD14 soluble CD14, LPS lipopolysaccharide, CXCL10 C-X-C motif chemokine 10, CXCR3 C-X-C motif chemokine receptor 3, IFN interferon, MPO myeloperoxidase; cccDNA covalently closed circular DNA, rcDNA relaxed circular DNA; Geq genome equivalent, TE transient elastography.

The primary outcomes of interest and study aim was to identify factors associated with markers of liver disease, which we measured as elevated liver enzymes and increased TE or Metavir score by liver biopsy. Spearman’s rank correlation coefficients for associations with liver inflammation (enzymes) and fibrosis are represented using a heat map (Fig 1 and S1 Fig). Results from regression analyses controlled for CD4+ T-cell count are shown in Table 2, and S3 and S4 Tables.

**Table 2:** Intrahepatic and circulating parameters associated with fibrosis outcomes by regression analysis (fibrosis in kPa by TE) and odds ratio (fibrosis by Metavir on biopsy), controlled by CD4+ T cell count.

Variable		Fibrosis kPa (TE)*				Fibrosis (Metavir)^†^	
		Linear Regression Coefficient (95% CI)	Exponentiated regression coefficient (95% CI)	Partial Eta-Squared (η2)	P-value	Odds Ratio (95% CI)	P-value
**Peripheral**	**Conjugated Bilirubin (mg/dL)**	1.00 (0.02, 1.98)	2.72 (1.02, 7.24)	0.11	0.045	0.24 (0.00, 86.15)	0.637
	**GGT (U/L)**	0.004 (0.002, 0.007)	1.004 (1.002, 1.007)	0.29	0.001	1.02 (1.00, 1.03)	0.063
	**AST (U/L)**	0.009 (0.003, 0.015)	1.009 (1.003, 1.016)	0.19	0.007	1.04 (1.00, 1.08)	0.080
	**LPS**^‡^ (pg./mL)	0.001 (-0.003, 0.006)	1.001 (0.997, 1.006)	0.01	0.589	1.02 (0.99, 1.05)	0.223
	**sCD14 log**_**10**_	-0.03 (-1.07, 1.00)	0.97 (0.34, 2.72)	0.00	0.950	1.58 (0.01, 451.69)	0.873
**Liver**	**CXCL10 (Δ-Δ-Ct)**^‡^	0.02 (0.01, 0.02)	1.02 (1.01, 1.02)	0.40	<0.001	1.10 (1.04, 1.16)	0.001
	**CXCR3 (Δ-Δ-Ct)**^‡^	0.15 (0.02, 0.28)	1.16 (1.02, 1.33)	0.14	0.026	3.52 (1.33, 9.26)	0.011
	**IFN-γ (Δ-Δ-Ct)**^§^	0.016 (-0.004, 0.035)	1.016 (0.996, 1.035)	0.08	0.112	1.06 (0.95, 1.18)	0.329
	**LPS (% area)**^**||**^	0.39 (0.01, 0.77)	1.48 (1.01, 2.15)	0.18	0.044	2.65 (0.27, 25.90)	0.401
	**HIV DNA**^¶^ **(copies/10**^**6**^ **cells)**	0.0007 (0.0000, 0.0015)	1.0007 (1.0000, 1.0015)	0.11	0.046	1.005 (1.000, 1.009)	0.034
	**CA-US HIV RNA**^¶^ **(copies/10**^**6**^ **18s)**	0.02 (-0.02, 0.05)	1.02 (0.98, 1.05)	0.03	0.283	1.47 (1.00, 2.17)	0.048
	HBV cccDNA** (copies/Geq)	0.18 (0.04, 0.31)	1.19 (1.04, 1.36)	0.23	0.013	12.06 (0.62, 234.70)	0.100
	HBV rcDNA** (copies/Geq)	0.002 (-0.001, 0.005)	1.002 (0.999, 1.005)	0.05	0.293	1.02 (1.00, 1.05)	0.018

*Linear regression analysis on log_e_ transformed TE adjusted for total CD4 count.

^†^Ordered logistic regression analysis on Metavir adjusted for total CD4 count.

n = 39, except where specified

^‡^n = 37

^§^n = 35

^||^n = 24

^¶^ = 38

**n = 27.

TE transient elastography, CI confidence interval, GGT γ-glutamyl transferase, AST Aspartate Aminotransaminase, CXCL10 C-X-C motif chemokine, CXCR3 C-X-C motif chemokine receptor 3, IFN interferon, LPS lipopolysaccharide, CA- cell associated, US unspliced, HIV human immunodeficiency virus, HBV hepatitis B virus, cccDNA covalently closed circular DNA, rcDNA relaxed circular DNA, Geq genome equivalent

Factors that had a statistically significant association with fibrosis are shaded in grey (indicating p<0.05) and include liver CXCL10 and liver CXCR3 mRNA. A weak but significant association was also seen with liver HIV DNA. Darker shading has been used to indicate factors that were significantly associated with both TE and liver biopsy (Metavir). Fibrosis kPa (TE) was log_e_ transformed due to skewness, therefore exponentiated regression coefficients are presented. For the exponentiated regression coefficient, (e.g. for liver CXCR3 with coefficient of 1.16): one unit increase in liver CXCR3 results in (1.16–1) x 100 = 16% percentage change in Fibrosis (TE). For the odds ratio, (e.g. for liver CXCR3 with odds ratio of 3.52): for one unit increase in liver CXCR3, the odds of F3 versus the combined F0 and F1 categories is 3.52 greater. Likewise, the odds of the combined F1 and F3 categories versus F0 is 3.52 greater. Partial eta squared values (η2) were obtained for the linear regression models to quantify the effect size, using Cohen’s benchmarks to categorise η2 as small (η2 = 0.01), medium (η2 = 0.06), and large (η2 = 0.14).

Intrahepatic mRNA for CXCL10 and CXCR3 were both strongly associated with liver fibrosis on regression analysis for TE (η2 = 0.40, p<0.001 and η2 = 0.14, p = 0.026) and liver biopsy (odds ratio 1.10 (1.04, 1.16), p = 0.001, odds ratio 3.52 (1.33, 9.26), p = 0.011 (Table 2)). A significant but weak association was seen between mRNA for CXCL10 and CXCR3 with HIV DNA.

A statistically significant association was found between liver LPS (% area) and TE (η2 = 0.18, p = 0.044, Table 2) but there was no evidence of an association with fibrosis by biopsy (Metavir). A weak but statistically significant association was also seen between intrahepatic HIV RNA and HBV rcDNA and liver fibrosis by biopsy (Metavir) (p = 0.048 and 0.018 and odds ratio (95% confidence interval—CI) of 1.47 (1.00, 2.17) and 1.02 (1.00, 1.05) respectively, Table 2) but there was no association with TE. We found no relationship between liver fibrosis and circulating markers of microbial translocation (peripheral LPS and sCD14), viral measures (circulating HIV or HBV) or inflammatory markers including CXCL10 and CCL2 (Fig 1, Table 2). Plasma HMGB1, a surrogate marker for cell death, was also not associated with liver fibrosis.

**Fig 2:**
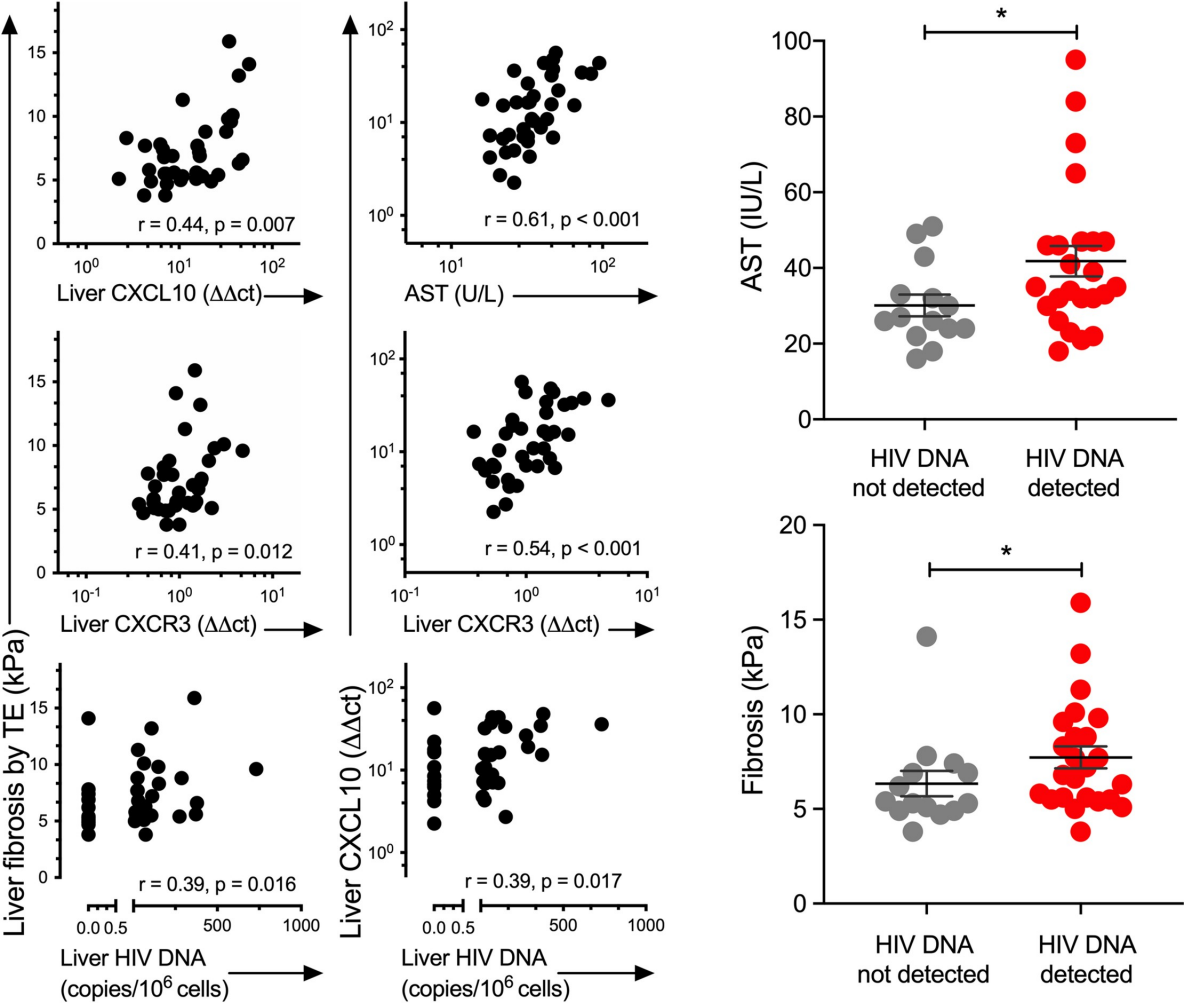
**(A) Liver fibrosis was significantly correlated with intrahepatic HIV DNA and with mRNA for CXCL10 and CXCR3.** Plots showing correlations between liver fibrosis by TE (kPa) with intrahepatic HIV DNA, CXCL10, CXCR3 (left side panels) and between liver CXCL10 levels with intrahepatic HIV DNA, CXCR3 and peripheral AST (right side panels). The lower limit of detection for HIV RNA and HIV DNA was one copy per well. If there was no HIV PCR signal, this was recorded as zero and if there was a detectable signal but <1, this was recorded as 0.5 copies. All data points were included in the Spearman correlation. r = Spearman rank correlation coefficient. HIV human immunodeficiency virus, CXCL10 C-X-C motif chemokine 10, CXCR3 C-X-C motif chemokine receptor 3, TE transient elastography, AST Aspartate transaminase. (B) Liver fibrosis (kPa) and AST were higher in participants with detectable HIV DNA in the liver. Each symbol represents values from each participant. The lines represent the median and IQR. Comparisons were made using Wilcoxon Rank Sum test (* p<0.05).

Liver enzymes can also be a marker of liver disease. We observed a positive correlation between liver enzymes (markers of liver inflammation) and liver fibrosis as expected (Fig 1, Fig 2). Intrahepatic mRNA for CXCL10 was significantly associated with ALT (η2 = 0.18, p = 0.009), AST (η2 = 0.30, p = 0.001) and GGT (γ-glutamyl transferase) (η2 = 0.13, p = 0.029) (regression analysis, S3 Table), whilst peripheral CXCL10 levels were significantly associated with GGT only (η2 = 0.13, p = 0.028, regression analysis, S3 Table). CD4+ T-cell associated US-HIV RNA was associated with ALP (alkaline phosphatase) (η2 = 0.50, p<0.001), GGT (η2 = 0.16, p = 0.016) and AST (η2 = 0.24, p = 0.003), S3 Table). Individual liver enzymes were also significantly associated with other parameters, but no other parameter associated with more than two liver enzymes (S3 Table).

### HIV DNA and RNA in the liver are related to adverse liver outcomes

HIV DNA and CA-US HIV RNA were detected less frequently in liver than blood. HIV DNA and CA-US HIV RNA were detected in blood CD4+ T-cells in 100% (39/39) and 84% (31/37) of participants respectively and in liver in 62% (24/38) and 45% (17/38) of participants respectively. The relationship between liver HIV DNA and liver fibrosis (TE and biopsy) was statistically significant but weak for TE (η2 = 0.11, p = 0.046) and the odds ratio very close to one for Metavir score (OR = 1.005, p = 0.034) (Table 2, Fig 2). Those with detectable HIV DNA had significantly higher levels of fibrosis (TE) than those in whom HIV DNA in the liver was not detectable (Fig 2B).

### Intrahepatic CXCL10 was located in hepatocytes and strongly associated with HIV DNA and RNA

**Fig 3:**
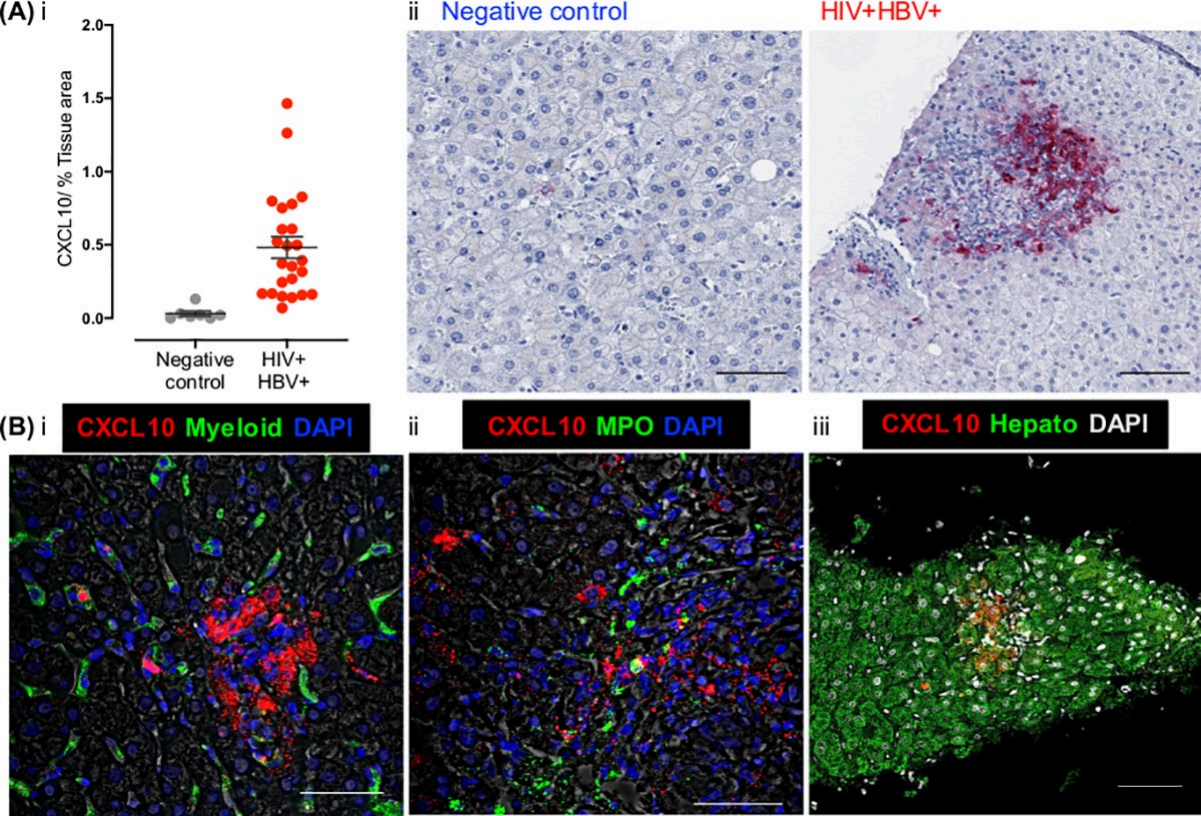
Intrahepatic CXCL10 staining is localised in hepatocytes and increased in HIV-HBV co-infected liver biopsies. (A) Biopsies from people living with HIV-HBV coinfection and controls who were HIV and HBV negative were examined using immunohistochemistry (IHC) for CXCL10 (red-AP). The percentage area positive for CXCL10 staining (dark pink stain) was quantified using Photoshop CS5 and Fovea tools. Comparisons were made using the Wilcoxon Rank Sum test (* p<0.05). (i) The median and IQR for percentage area positive for CXCL10 is shown as well as (ii) representative pictures. CXCL10 staining was found within inflammatory Infiltrates, close to portal regions or blood vessels. (B) Fluorescence microscopy was performed using an RNAscope approach with a probe targeting CXCL10 (red), a nuclear stain DAPI (blue or grey) and antibodies to either (i) Myeloid cells (CD68+CD163), (ii) myeloperoxidase (MPO) or (iii) Hepatocyte (green). This demonstrates CXCL10 is predominantly located in hepatocytes. Scale bars: 100𝜇m.

Given the strong relationship between intrahepatic CXCL10 and fibrosis, we were interested in determining which cells were producing CXCL10. Immunohistochemistry demonstrated CXCL10 expression in liver biopsy sections in association with inflammatory infiltrates in the portal regions in liver biopsies from people living with HIV-HBV co-infection but there was minimal expression of CXCL10 in healthy controls (Fig 3). To determine the cellular location of CXCL10 we performed co-staining to identify CD4+ and CD8+ T-cells, NK cells (NKp44+NKp46+CD57+), myeloid cells (CD68+ CD163+) neutrophils (MPO+) and hepatocytes (MA5-12417-OCH1E5). CXCL10 was predominantly located in hepatocytes (Fig 3). Low CXCL10 expression was seen in myeloid cells (CD68+CD163+), and neutrophils (MPO+), and in a few CD8+ T-cells that were also positive for CXCL10. There was no CXCL10 staining with CD4+ T-cells or NK (CD56+) cells.

Levels of liver CXCL10 (quantified by PCR) were associated with intrahepatic HIV DNA (r = 0.21; p = 0.019) and peripheral HIV RNA (r = 0.21, p = 0.018) (Figs 1 and 3, S4 Table). mRNA for the CXCL10-receptor CXCR3, was also measured in the liver and was strongly associated with levels of both CXCL10 (p<0.001) and HIV DNA (p<0.001) in the liver (Fig 2, S4 Table, S2 Fig). IFN-α and β were not detected in liver samples by PCR (S2 Table) whilst IFN-γ levels were strongly and significantly correlated with intrahepatic mRNA for CXCL10 and CXCR3 (Fig 1, S4 Table) and intrahepatic HIV DNA (S2 Fig, S4 Table (p = 0.003, regression analysis).

Together these data suggest a very significant but likely complex relationship between intrahepatic CXCL10, CXCR3, IFN-γ and HIV DNA. In addition, intrahepatic mRNA for CXCL10 and CXCR3 were also associated with liver fibrosis and inflammation (Table 2, S3 Table).

### Microbial Translocation

We next examined the relationship between microbial translocation and monocyte activation with liver fibrosis and inflammation. Intrahepatic LPS (% area) was significantly associated with liver fibrosis by TE (η2 = 0.18, p = 0.044) but not liver biopsy (Table 2). Intrahepatic LPS (% area) was also associated with liver HIV DNA (η2 = 0.32, p = 0.006), and with sCD14 (η2 = 0.18, p = 0.042), S4 Table).

sCD14 and plasma LPS were not associated with liver fibrosis. Plasma levels of LPS were associated with ALP and with GGT (η2 = 0.24, p = 0.003 and η2 = 0.15, p = 0.018, respectively), regression analysis, S3 Table). sCD14 was associated with liver LPS (see above) and with measurements of HIV, including plasma HIV RNA, and CD4+ T-cell HIV DNA (S3 Fig). These relationships were significant on regression analysis and have been well described previously [43].

### HIV infection, with IFN-γ and P3CSK4, leads to a significant increase in CXCL10 production from HBV-infected hepatic cell lines

Based on our finding that intrahepatic CXCL10 was in hepatocytes and was associated with adverse liver-related outcomes and increased intrahepatic HIV DNA, HIV RNA and IFN-γ, we hypothesised that HIV infection of hepatocytes in the setting of inflammation and elevated IFN-γ, may drive increased production of CXCL10 and inflammation. We tested this hypothesis in vitro using the HBV-transfected AD38 hepatocyte cell line and the parental non-transfected HepG2 cell line.

We previously found that IFN-γ and LPS (containing a mixture of TLR-2 and 4 agonists (Sigma-Aldrich)), resulted in a significant increase in CXCL10 production following stimulation of hepatocyte cell lines [9]. In contrast, purified LPS (LPS-EB, TLR4 agonist (InvivoGen, San Diego, USA)) with IFN-γ did not increase CXCL10 production above either alone, suggesting an effect of TLR2 alone. There was a significant increase in CXCL10 in both cell lines in response to IFN-γ/P3CSK4 (TLR 1–2 agonist) which was therefore used in subsequent experiments.

**Fig 4:**
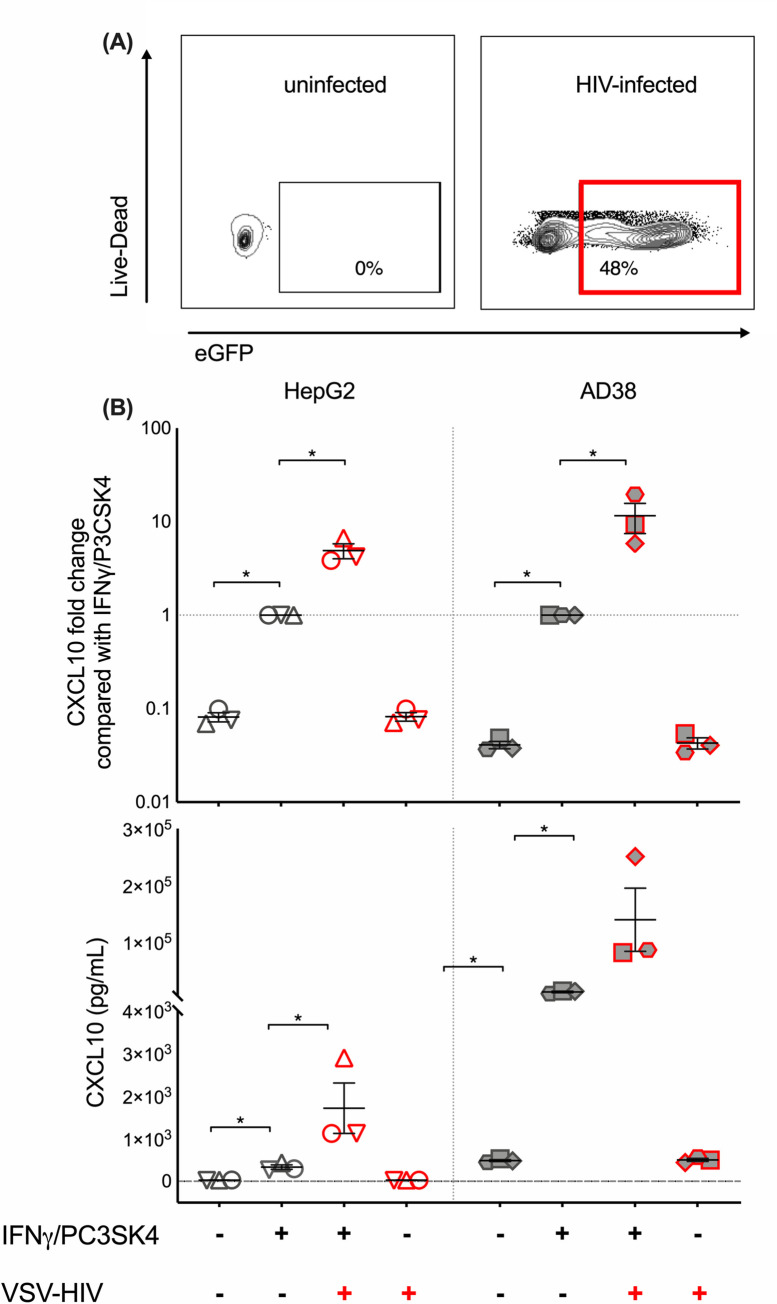
CXCL10 production is increased with HIV infection and is higher in HBV-producing cell lines. (A) A representative flow cytometry plot showing eGFP expression (indicating infection with VSV-G-pseudotyped NL4.3Δenv eGFP HIV (MOI 0.5)) in live cells after gating on singlets and live cells, in uninfected hepatocytes (left plot) and hepatocytes infected with VSV-G-pseudotyped NL4.3Δenv eGFP HIV (MOI 0.5). (B) CXCL10 production measured by ELISA following stimulation of HepG2 (open symbols), and HBV-producing AD38 (filled symbols) hepatocyte cell lines with 500ng/ml IFN-γ plus1000ng/ml P3CSK4 and infected with VSV G-pseudotyped NL4.3Δenv eGFP HIV (MOI 0.5). HIV infection is indicated by symbols having a red border. Individual symbols represent the mean of replicates from a single experiment. The median+/-SEM for each stimulus from multiple experiments is shown. Comparisons between conditions were made using Wilcoxon Rank Sum test (* p<0.05). VSV-G-pseudotyped NL4.3Δenv eGFP HIV Vesicular stomatitis virus (VSV) glycoprotein G-pseudotyped NL4.3 virus with an envelope deletion expressing green fluorescent protein, MOI multiplicity of infection, HIV human immunodeficiency virus, CXCL10 C-X-C motif chemokine 10, IFN interferon, P3CSK4 Pam3CysSerLys4.

**Fig 5:**
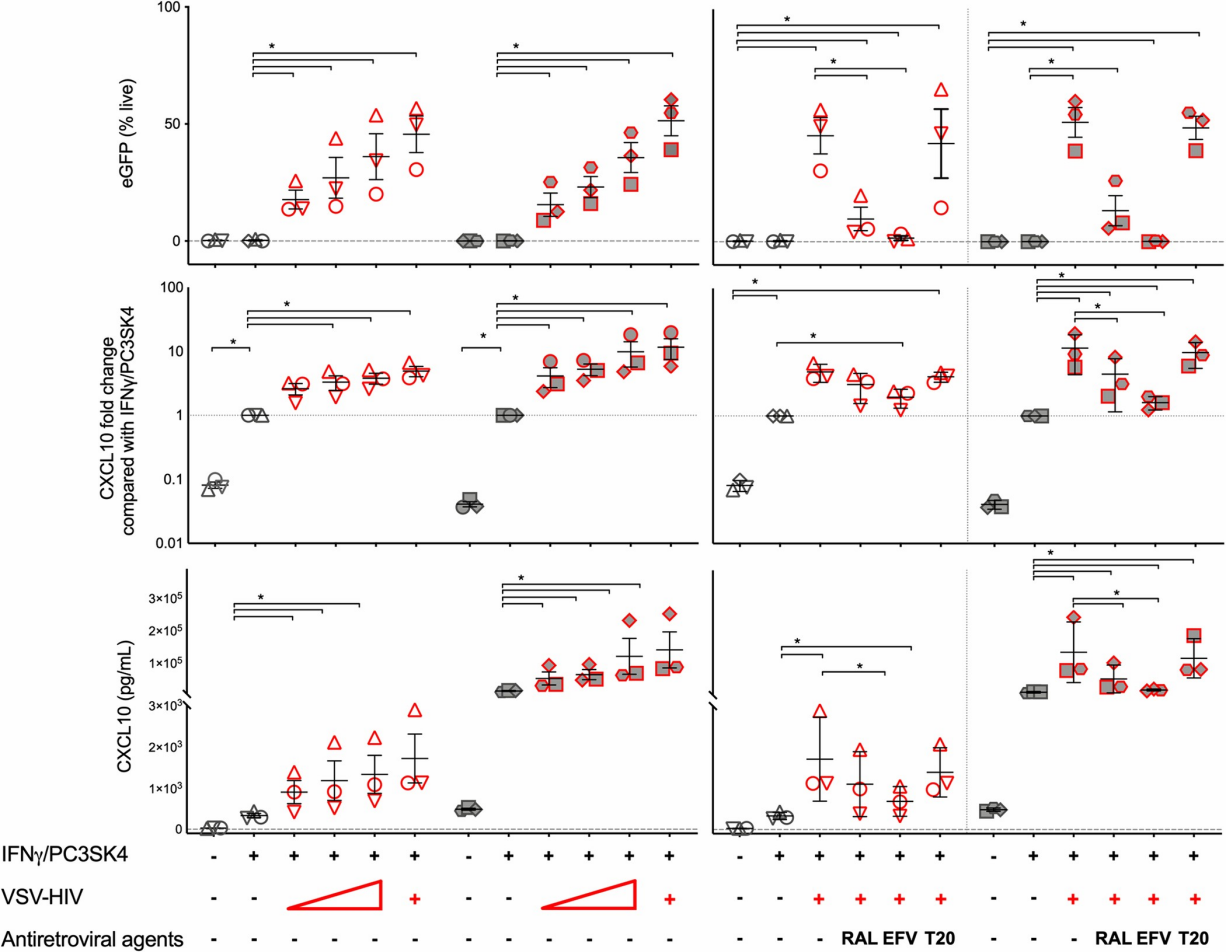
CXCL10 production is enhanced in the setting of HIV and HBV co-infection in vitro and decreased with the addition of antiretroviral agents efavirenz or raltegravir. CXCL10 production was measured by ELISA (absolute and fold change, middle and lower panel) and eGFP expression measured by flow cytometry (upper panel) following stimulation of HepG2 (open symbols), and HBV-producing AD38 (filled symbols) cells, with 500ng/ml IFN-γ plus1000ng/ml P3CSK4 and infected with VSV-G-pseudotyped NL4.3Δenv eGFP HIV. The left sided panels show eGFP expression and CXCL10 production after cells were stimulated with 500ng/ml IFN-γ plus1000ng/ml P3CSK4 and infected with VSV-G-pseudotyped NL4.3Δenv eGFP HIV with a MOI of 0.0625, 0.125, 0.25 and 0.5. Increasing MOI is indicated by the triangle at the bottom of the Fig. The right sided panels show these same parameters, following infection with VSV-G-pseudotyped NL4.3Δenv eGFP HIV (red border) with MOI of 0.5 in the presence and absence of the antiretroviral agents efavirenz (EFV), raltegravir (RAL) and the fusion inhibitor T20. Individual symbols represent the mean of replicates from a single experiment. The median+/-SEM for each stimulus from multiple experiments is shown. Comparisons between conditions were made using Wilcoxon Rank Sum test (* p<0.05). HIV human immunodeficiency virus, CXCL10 C-X-C motif chemokine 10, GFP green fluorescent protein, IFN interferon, VSV-G-pseudotyped NL4.3Δenv eGFP HIV Vesicular stomatitis virus (VSV) glycoprotein G-pseudotyped NL4.3 virus with an envelope deletion expressing green fluorescent protein, MOI multiplicity of infection, P3CSK4 Pam3CysSerLys4, efavirenz (EFV), raltegravir (RAL).

To determine the effects of HIV infection on CXCL10 production, we infected both hepatocyte cell lines with VSV-G-NL4.3Δenv-eGFP HIV in combination with IFN-γ/P3CSK4. This allowed for high level consistent single round infection of target cells; and infected cells were easily identified as GFP positive (Fig 4). We found that the constitutive expression of CXCL10 was significantly higher in the HBV-transfected AD38 compared to the uninfected HepG2 cell line. Infection with VSV-G-NL4.3Δenv-eGFP HIV significantly increased production of CXCL10 by both HepG2 and AD38 cells in the presence of IFN-γ/P3CSK4 but there was no increase in CXCL10 following VSV-G-NL4.3Δenv-eGFP HIV infection alone (Fig 4). While the absolute values of CXCL10 were far higher in the HBV-producing AD38 cells than HepG2 cells, the fold increase in CXCL10 production in the presence of HIV, in both HBV uninfected and HBV-infected cell lines was similar (Fig 4, Fig 5). Absolute values also varied between experiments for the same cell line, however the fold change was consistent. There was a clear dose effect of HIV infection with increasing CXCL10 production with an increasing multiplicity of infection (MOI) in both cell lines (Fig 5). To demonstrate that this effect was not secondary to eGFP and was specific to HIV infection, we performed the same experiments using a VSV-G pseudotyped virus that expresses luciferase and has a deletion in envelope and vpr (VSV.G-NL4-3-luciferase-Δenv-Δvpr). An increase in CXCL10 production with increasing MOI was again seen and this increase was statistically significant in HBV-producing AD38 cells (S4 Fig).

To confirm the impact of HIV infection on CXCL10 production, we repeated the infections in the presence of antiretroviral agents. We used three antiretrovirals, the non-nucleoside reverse-transcriptase inhibitor (NNRTI) efavirenz, the integrase inhibitor raltegravir and the entry inhibitor T20 (Fig 5). These antiretroviral agents are active against HIV but are not active against HBV. Both efavirenz and raltegravir significantly reduced eGFP expression demonstrating successful inhibition of HIV infection. However, whilst efavirenz abolished eGFP expression after infection, the addition of raltegravir led to only a partial reduction in eGFP expression. The persistent partial eGFP expression was likely due to unintegrated viral DNA, consistent with previous reports [44].

There was a significant reduction in CXCL10 production with both ARVs, suggesting that CXCL10 production was dependent on virus integration or downstream steps of virus replication. A reduction in CXCL10 was observed following ARV treatment of both the HBV-producing AD38 and the parent HepG2 hepatocyte cell lines. The HIV gp41 fusion inhibitor T20 had no effect on CXCL10 production nor on eGFP expression as expected, given that the VSV-G receptor bypasses fusion mediated events leading to HIV infection. The addition of HBV proteins (HBV core or HBsAg protein) or HIV (gp120 or ssHIV RNA) also had no effect on CXCL10 production (S5 Fig).

Together, these data demonstrate that HIV infection of hepatocytes, in the presence of IFN-γ/P3CSK4, leads to a significant increase in CXCL10 production, in both the HBV-transfected and parental hepatocyte cell line. These effects were inhibited with an NNRTI and partially inhibited by an integrase inhibitor suggesting that the enhanced production of CXCL10 was a consequence of events post HIV integration and was not due to an effect of the VSV-G protein.

## Discussion

Understanding the drivers of accelerated liver disease in HIV-HBV co-infection remains of high importance, despite the availability of HBV-active ART. This is the first study to systematically assess inflammatory markers such as CXCL10 as well as HIV DNA and RNA in matched liver and blood samples and determine the relationship of intrahepatic inflammation and HIV to clinical outcomes. We show that in untreated HIV-HBV co-infected individuals, while there were multiple associations observed, both liver fibrosis and liver enzyme abnormalities were significantly associated with increased intrahepatic mRNA for CXCL10 and its receptor CXCR3 and that expression of CXCL10 was primarily in hepatocytes. We then demonstrated in an in vitro model that HIV infection of HBV-infected cells can significantly increase production of CXCL10. Together these data clearly demonstrate a key relationship between fibrosis, intrahepatic CXCL10 and HIV infection.

The exact mechanism of how HIV drives production of CXCL10 from hepatocytes is unclear. We found that HBV-infected compared to uninfected hepatocyte cell lines produced significantly more CXCL10 in response to IFN-γ/P3CSK4 and this was further increased with HIV infection. Previous work in two separate models—HIV-HCV co-infection [45] and HIV infection of astrocytes [46]—demonstrated that the HIV tat protein, a potent transcriptional activator, can directly induce CXCL10 expression. In our model, HIV alone did not increase CXCL10 expression. Rather, a significant increase in CXCL10 following HIV infection was only observed in the presence of IFN-γ/P3CSK4, which we included to mimic inflammation from microbial products. We propose that HIV infection, potentially through tat, acts to enhance/potentiate the effect of IFN-γ/P3CSK4 on increasing production of CXCL10 from hepatocytes.

**Fig 6:**
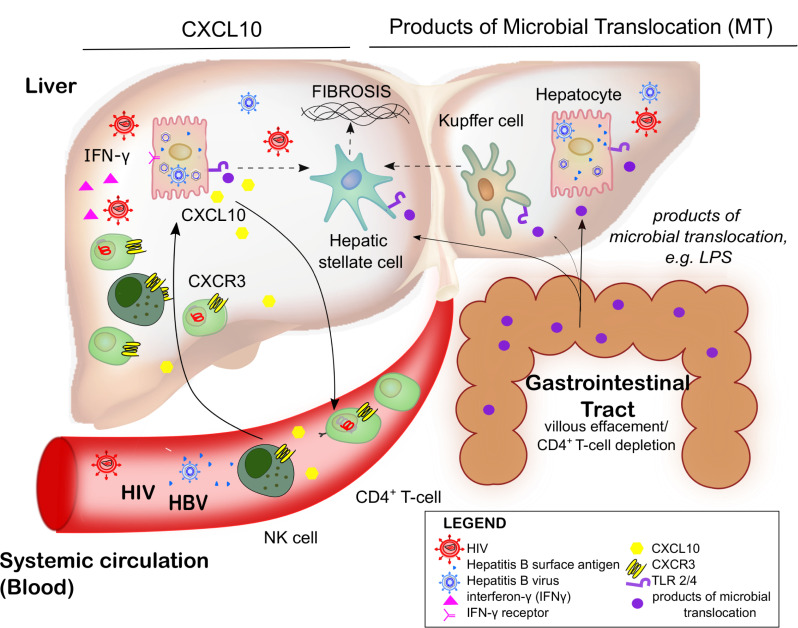
Factors driving liver disease pathogenesis in HIV-HBV co-infection In HIV-HBV co-infection we propose a model where liver fibrosis is driven by an increase in production of CXCL10 by hepatocytes (left hand side of diagram) and products of microbial translocation (right hand side of diagram). Hepatocytes produce CXCL10 and production is enhanced following infection with HBV and HIV in the presence of IFN-γ. CXCL10 recruits activated T-cells and NK cells that express CXCR3 to the liver. In the absence of antiretroviral therapy, this is an ideal environment for HIV replication which produces IFN-γ and drives further CXCL10 production. In addition, altered GI tract permeability in the setting of HIV infection leads to an increase in circulating microbial products, including LPS which binds to TLR-4 and P3S4K4 which binds to TLR-2 expressed on hepatocytes, Kupffer cells and hepatic stellate cells. This cycle of CXCL10-mediated inflammation, together with elevated LPS directly activates hepatic stellate cells (HSC) driving fibrosis. HIV human immunodeficiency virus, HBV hepatitis B, CXCL10 C-X-C motif chemokine 10, IFN interferon, NK natural killer, CXCR3 C-X-C motif chemokine receptor 3, GI gastrointestinal tract, LPS lipopolysaccharide, TLR toll like receptor, HSC hepatic stellate cells.

We demonstrated that CXCL10 production from hepatocytes increases following direct infection with HIV. In vivo, we also showed that CXCL10 production in HIV-HBV co-infection was localised to areas of inflammatory infiltration in the periportal regions of liver tissue, but was almost entirely located in hepatocytes, rather than in the infiltrating lymphocytes or neutrophils. Whether CXCL10 production in vivo primarily comes from HIV-infected or uninfected hepatocytes still warrants further investigation. However, enhanced CXCL10 production by hepatocytes could lead to recruitment of CXCR3+ T-cells or NK cells to the liver. Activated CXCR3+ CD4+ T-cells are highly permissive for HIV infection [47] and both activated T-cells and NK cells are potent producers of IFN-γ which would further increase CXCL10 production from hepatocytes [48]. Together this would set up a cycle of more CXCL10 and more HIV infection (Fig 6). Non-parenchymal cells may also be important as a source of interferon, leading to the induction of CXCL10 from hepatocytes, as has been demonstrated in HCV infection [15].

CXCL10 has been associated with liver inflammation and fibrosis across a range of etiologies [13] and mediates this effect through various mechanisms. First, through recruitment of CXCR3+ NK cells. In chronic HBV infection, NK cells have been implicated in tumour necrosis factor-related apoptosis-inducing ligand (TRAIL)-mediated hepatocyte death [17]. CXCL10 can also mediate hepatocyte apoptosis directly by binding TLR-4 and inducing protein kinase B and Jun N-terminal kinase activation, leading to apoptosis by cleavage of caspase-8, caspase-3 and p-21 activated kinase 2 [49]. We did not find an association between fibrosis levels and circulating levels of HMGB1, a marker of cell death, but liver biopsy specimens were not stained for HMGB1 nor markers of apoptosis in this study. However, our previous work has shown increased apoptosis in liver biopsies in HIV-HBV co-infection compared with HBV mono-infection [50].

We observed clear differences in the relationship of intrahepatic and blood HIV DNA and RNA with multiple clinical endpoints, suggesting that in the liver, HIV replication is compartmentalised. What remains unclear is where that HIV replication is predominantly occurring. In this study, intrahepatic HIV DNA and RNA were quantified in unsorted liver biopsies but in blood, we quantified HIV in sorted CD4+ T-cells. We were therefore unable to demonstrate that the intrahepatic HIV DNA and RNA was in T-cells. Other cells in the liver could also be infected with HIV, such as Kupffer cells, hepatic stellate cells or hepatocytes. We found that sCD14 (a marker of monocyte activation) was associated with multiple markers of HIV–in both the periphery and in the liver, however this is not direct proof of Kupffer cell infection. Defining the specific cells where HIV resides in liver is needed and could potentially be addressed using fluorescent probes as recently demonstrated with RNA and DNA scope [51].

Most international guidelines now recommend immediate ART for all people living with HIV so our findings may have less relevance to individuals on HBV-active ART that suppresses HIV and HBV replication. However, we propose that CXCL10 remains an important mediator of liver disease in HIV-HBV co-infection, even in the setting of ART. First, our previous work has clearly demonstrated that CXCL10 remains elevated on ART [9]. Second, we show here that the effects of HIV infection of hepatocyte cell lines on CXCL10 production were mediated post HIV integration. It is well described that HIV can infect hepatocytes [20, 22, 52] and whether long-lived latent infection of these cells can be established remains unclear. Finally, recent data demonstrates that the level of HBV DNA prior to initiation of ART was associated with long term clinical outcomes including liver disease on ART [53] implying a long lasting impact of virus replication prior to ART, even following virological control.

This is the first study of people living with HIV and HBV with matched liver biopsy and blood specimens and recruitment from a single site in a resource limited setting. Analysis of biopsy specimens provides an important and increasingly rare opportunity to understand liver disease pathogenesis. Limitations included the cross-sectional design, so we were only able to identify associations, which does not imply causation. We did however test our hypothesis for the cause of elevated CXCL10 using an in vitro model. We used hepatoma cell lines with stably transfected HBV, rather than primary hepatocytes or sodium-taurocholate transporting polypeptide receptor-enriched cell lines. We also used a VSV-G pseudotyped virus to achieve artificially high levels of HIV infection, given prior experiments with wild type NL4.3 showed no significant infection of hepatocyte cell lines [54]. Inhibition of the effect of HIV by the addition of antiretroviral agents that inhibit HIV reverse transcription or integration, confirmed a role for HIV infection (rather than the VSV-G protein) in driving CXCL10 production from hepatocyte cell lines. HIV infection of hepatocytes has been observed in vitro but is infrequent [20, 52].

Factors such as age, sex and alcohol consumption are well known to influence the development of liver fibrosis. These clinical parameters were available, however as the population was mainly young men with low alcohol intake, and neither alcohol, sex nor age were associated with fibrosis at the univariate level, we did not include these factors in the regression model. Low participant numbers also meant that we were limited in the number of factors that we could control for in our model.

Finally, we did not include control groups with untreated HBV or HIV mono-infection, as our goal here was to define factors associated with adverse liver outcomes in a population with HIV-HBV co-infection. Comparisons would be useful; however matching such cohorts is difficult given that age of acquisition of HBV is important for likelihood of clearance and liver disease and this differs in HIV-infected and uninfected individuals [55, 56].

We conclude that in people living with HIV and HBV, liver disease is associated with elevated intrahepatic mRNA for CXCL10 and CXCR3. In addition, CXCL10 was strongly associated with intrahepatic HIV DNA and RNA and not markers of HBV replication. We demonstrated that co-infection of hepatocytes in vitro with HIV and HBV significantly increased production of CXCL10. We propose a model whereby CXCL10 production is enhanced following HIV and HBV infection of hepatocytes, which can attract activated CXCR3+ NK and T-cells into the liver leading to enhanced HIV infection of T-cells, inflammation and development of fibrosis over time. HBV-active ART will reduce this cycle through inhibition of both HIV and HBV replication, however given CXCL10 remains elevated on ART, this pathway may also be important in driving liver disease on treatment and requires further investigation.

## Supporting information

S1 Fig Heat Map representing Spearman correlations between viral and inflammatory factors measured in subjects’ peripheral (blood/plasma) and liver samples. (TIF)

S2 Fig Relationship between intrahepatic HIV DNA and CA-US HIV RNA and liver outcomes. (TIF)

S3 Fig sCD14 was associated with HIV DNA in CD4+ T cells, plasma HIV RNA and peripheral CXCL10 as well as inversely associated with nadir CD4+ T cell count. sCD14 soluble CD14, CXCL10 C-X-C motif chemokine 10.
(TIF)

S4 Fig CXCL10 production is increased from AD38 cells with VSV.g-NL4-3-Luc integration at MOI 1. Increasing MOI is indicated by the triangle at the bottom of the Fig and represents MOI from 0.0625–1.0.HIV human immunodeficiency virus, CXCL10 C-X-C motif chemokine 10, IFN interferon, VSV-G pseudotyped virus that expresses luciferase and has a deletion in envelope and vpr (VSV.G-NL4-3-luciferase-Δenv-Δvpr), MOI multiplicity of infection, P3CSK4 Pam3CysSerLys4, efavirenz (EFV).
(TIF)

S5 Fig The addition of HBV proteins (HBV core or HBsAg protein) or HIV (gp120 or ssHIV RNA) did not further increase CXCL10. CXCL10 C-X-C motif chemokine 10, IFN interferon, P3CSK4 Pam3CysSerLys4, HBV Hepatitis B virus, sAg surface antigen, cAg core antigen, ssRNA single stranded HIV RNA, gp120 glycoprotein 120, HIV human immunodeficiency virus.
(TIF)

S1 Table Primer sequences for quantitative RT-PCR. C-X-C motif chemokine 10 (CXCL10), Large Ribosomal Protein (RPLPO), interferon (IFN)
(DOCX)

S2 Table Immune activation, apoptosis and viral parameters All values are presented as median (25th-75th percentiles) unless otherwise stated; n = 39, except where specified: ^‡^n = 37, ^§^n = 35, ^||^n = 24, ^||^ = 38, **n = 27 HMGB1 high mobility group box-1, CCL-2 C-C motif chemokine 2, sCD14 soluble CD14, LPS lipopolysaccharide, IHC immunohistochemistry, CXCL10 C-X-C motif chemokine, pg. picograms, IHC immunohistochemistry, CXCR3 C-X-C motif chemokine receptor 3, IFN interferon, CA- cell associated, US unspliced, HIV human immunodeficiency virus, HBV hepatitis B virus, cccDNA covalently closed circular DNA, rcDNA relaxed circular DNA; Geq genome equivalent. (DOCX)

S3 Table Factors associated with liver enzymes by regression analysis on loge transformed outcome, or log10 transformed (plasma sCD14) adjusted for total CD4 count. Factors that had a statistically significant association with fibrosis are shaded in grey (indicating p<0.05). Darker shading has been used to indicate factors that were significantly associated with at least three liver enzymes. Partial eta squared values (η2) were obtained for the linear regression models to quantify the effect size, using Cohen’s benchmarks to categorise η2 as small (η2 = 0.01), medium (η2 = 0.06), and large (η2 = 0.14). n = 39, except where specified: ^‡^n = 37, ^§^n = 35, ^||^n = 24, ^¶^n = 38, **n = 27 CI = Confidence Interval, ALP alkaline phosphatase, GGT γ-glutamyl transferase, ALT Alanine transaminase, AST Aspartate transaminase, CXCL10 C-X-C motif chemokine, pg. picograms, CXCR3 C-X-C motif chemokine receptor 3, IFN interferon, LPS lipopolysaccharide, HIV human immunodeficiency virus, HBV hepatitis B virus, CA- cell associated, cccDNA covalently closed circular DNA, rcDNA relaxed circular DNA, Geq genome equivalent.
(DOCX)

S4 Table Factors associated with hepatic inflammatory markers CXCL10, CXCR3, IFN-γ, LPS % area and circulating sCD14 by regression analysis on log_e_ transformed outcome, or log_10_ transformed (plasma sCD14) adjusted for total CD4 count. Factors that had a statistically significant association with fibrosis are shaded in grey (indicating p<0.05). Partial eta squared values (η2) were obtained for the linear regression models to quantify the effect size, using Cohen’s benchmarks to categorise η2 as small (η2 = 0.01), medium (η2 = 0.06), and large (η2 = 0.14). n = 39, except: ^‡^n = 37, ^§^n = 35, ^||^n = 24, ^¶^ n = 38, **n = 27, ^††^n = 33, ^‡‡^n = 22, ^§§^n = 23, ^||||^n = 32, ^¶^^¶^n = 26; CI = Confidence Interval, CXCL10 C-X-C motif chemokine, CXCR3 C-X-C motif chemokine receptor 3, IFN interferon, LPS lipopolysaccharide, sCD14 soluble CD14, HIV human immunodeficiency virus, HBV hepatitis B virus, CA- cell associated, US unspliced; rcDNA relaxed circular DNA, Geq genome equivalent.
(DOCX)
