# Supplementary figures and images for: Intrahepatic CXCL10 is strongly associated with liver fibrosis in HIV-Hepatitis B co-infection
Source: PLoS Pathog. 2020 Sep 8;16(9):e1008744. doi: 10.1371/journal.ppat.1008744 (PMC7521747; doi:10.1371/journal.ppat.1008744)

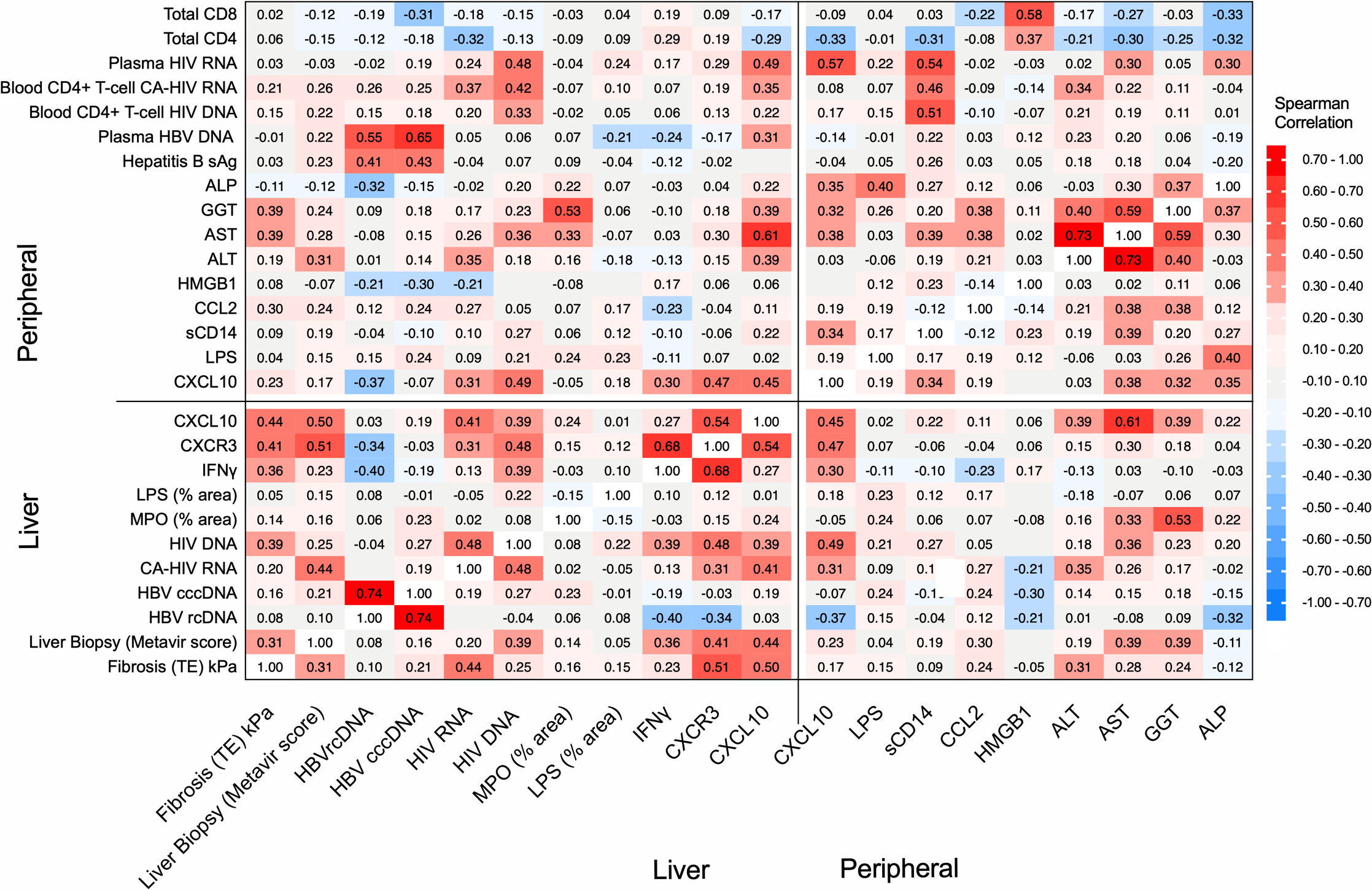

Supplement: S1 Fig — (TIF) [file ppat.1008744.s001.tif]

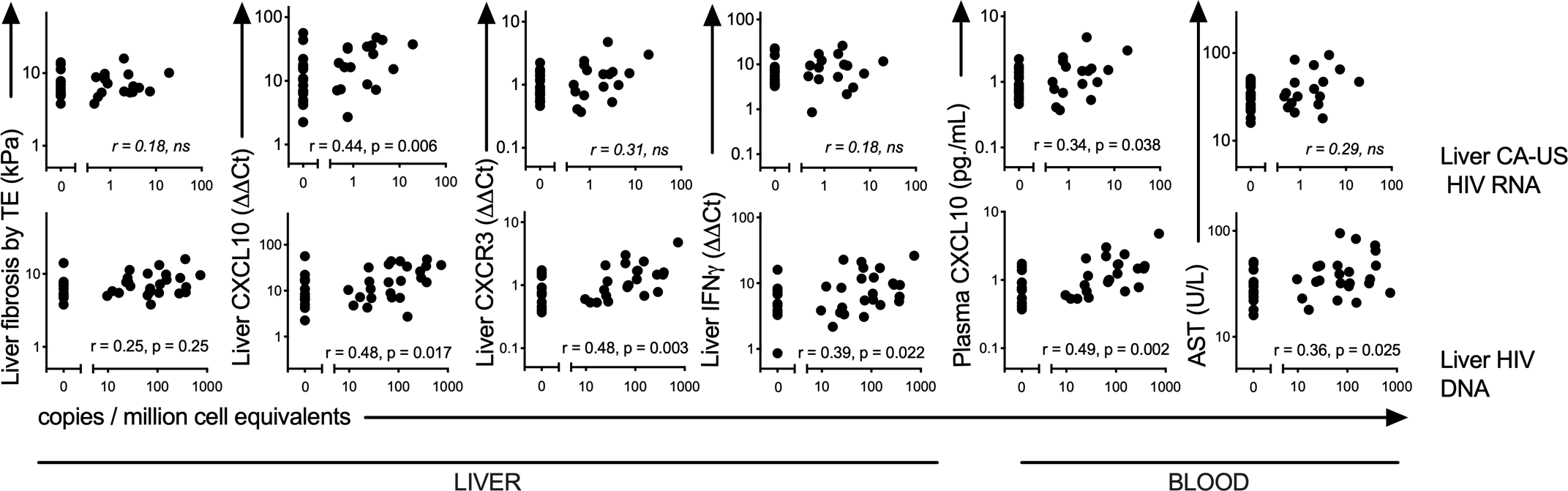

Supplement: S2 Fig — (TIF) [file ppat.1008744.s002.tif]

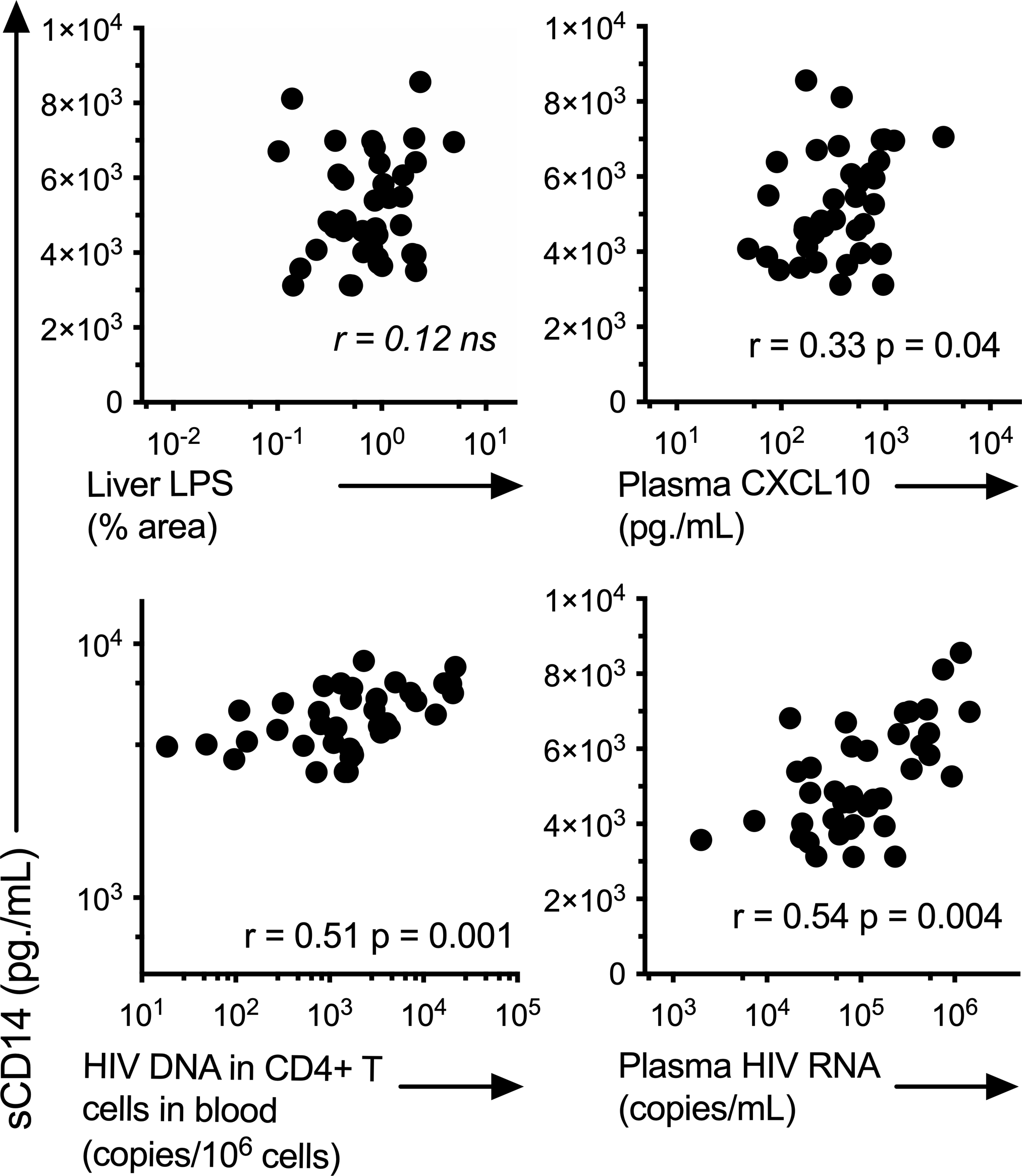

Supplement: S3 Fig — sCD14 soluble CD14, CXCL10 C-X-C motif chemokine 10. (TIF) [file ppat.1008744.s003.tif]

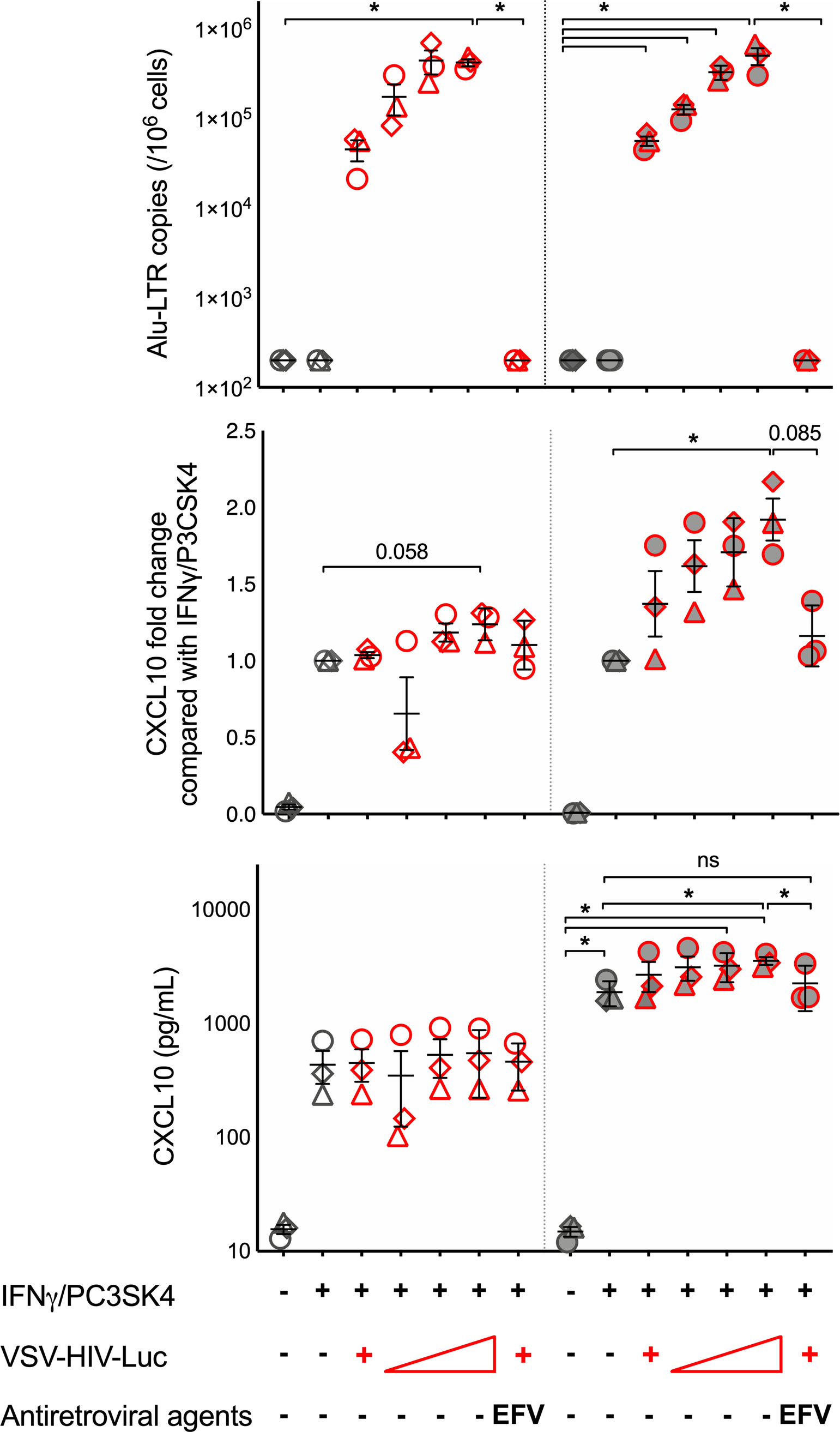

Supplement: S4 Fig — Increasing MOI is indicated by the triangle at the bottom of the Fig and represents MOI from 0.0625–1.0.HIV human immunodeficiency virus, CXCL10 C-X-C motif chemokine 10, IFN interferon, VSV-G pseudotyped virus that expresses luciferase and has a deletion in envelope and vpr (VSV.G-NL4-3-luciferase-Δenv-Δvpr), MOI multiplicity of infection, P3CSK4 Pam3CysSerLys4, efavirenz (EFV). (TIF) [file ppat.1008744.s004.tif]

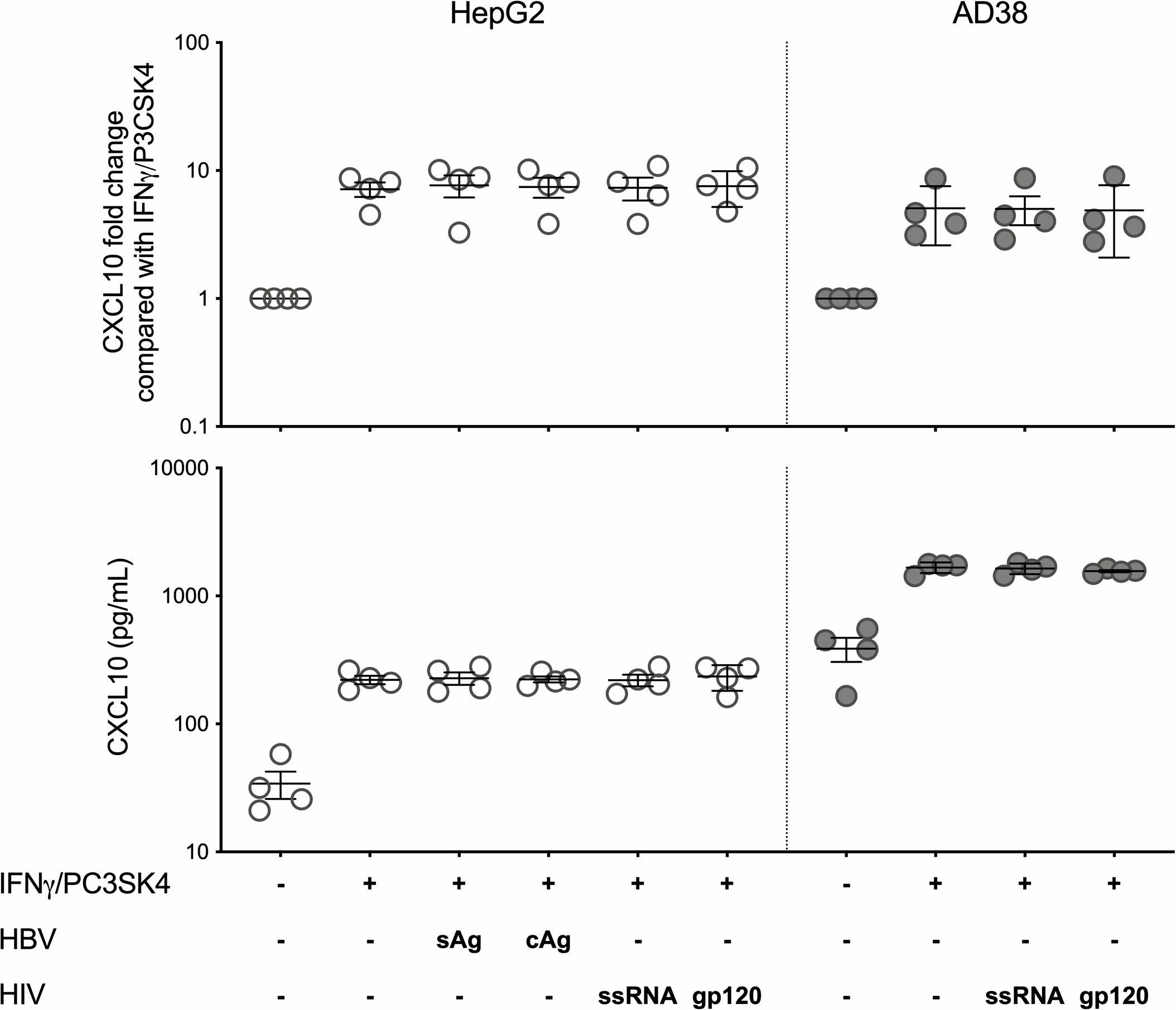

Supplement: S5 Fig — CXCL10 C-X-C motif chemokine 10, IFN interferon, P3CSK4 Pam3CysSerLys4, HBV Hepatitis B virus, sAg surface antigen, cAg core antigen, ssRNA single stranded HIV RNA, gp120 glycoprotein 120, HIV human immunodeficiency virus. (TIF) [file ppat.1008744.s005.tif]
