# Supplementary material for: Intrahepatic CXCL10 is strongly associated with liver fibrosis in HIV-Hepatitis B co-infection
Source: PLoS Pathog. 2020 Sep 8;16(9):e1008744. doi: 10.1371/journal.ppat.1008744 (PMC7521747; doi:10.1371/journal.ppat.1008744)
Supplement: S1 Table — C-X-C motif chemokine 10 (CXCL10), Large Ribosomal Protein (RPLPO), interferon (IFN) (DOCX) [file ppat.1008744.s006.docx]

#### S1 Table. Primer sequences for quantitative RT-PCR

| **Target** | **Primer 1** | **Primer 2** |
| --- | --- | --- |
| HIV RNA clade A/E 1^st^ round | **5’-CTGGGTCTCTCTDGTTAGAC-3’** | **5’- CCATCTCTCTCCTTCTAGC-3’** |
| HIV RNA clade A/E 2^nd^ round | **5’- GGTAACTAGAGATCCCTCAG-3’** | **5’- CCATCTCTCTCCTTCTAGC-3’** |
|  |  |  |
| CXCL10 cDNA | **5’-CCAATTTTGTCCACGTGTTG-3’** | **5’-TTCTTGATGGCCTTCGATTC-3’** |
| RPLPO control | **5'-AGATGCAGCAGATCCGCAT-3'** | **5'- GATGGCCTTGCGCA-3'** |
|  |  |  |
| IFN-α | **5’-AATGGCCTTGACCTTTGCTT-3’** | **5’CACAGAGCAGCTTGACTTGC-3’** |
| IFN-β | **5’-CGACACTGTTCGTGTTGTCA-3’** | **5’- GAAGCACAACAGGAGAGCAA-3’** |
| IFN-γ | **5’- GGCATTTTGAAGAATTGGAAAG-3’** | **5’- TTTGGATGCTCTGGTCATCTT-3’** |

C-X-C motif chemokine 10 (CXCL10), Large Ribosomal Protein (RPLPO), interferon (IFN)
