# Supplementary material for: Intrahepatic CXCL10 is strongly associated with liver fibrosis in HIV-Hepatitis B co-infection
Source: PLoS Pathog. 2020 Sep 8;16(9):e1008744. doi: 10.1371/journal.ppat.1008744 (PMC7521747; doi:10.1371/journal.ppat.1008744)
Supplement: S2 Table — (DOCX) [file ppat.1008744.s007.docx]

#### S2 Table. Immune activation, apoptosis and viral parameters

|  | % Samples positive (detected) | Values (median (25-75^th^ centile) |
| --- | --- | --- |
| **Plasma** |  |  |
| Plasma HMGB1, ng./mL | All (100%) | 3.08 (2.05-4.51) |
| Plasma CCL-2, pg./mL^‡^ | All (100%) | 185.6 (84.9-271.57) |
| Plasma sCD14, log10 pg./mL | All (100%) | 3.68 (3.60-3.81) |
| Plasma LPS, pg./mL^‡^ | All (100%) | 50.6 (36.9-83.6) |
| Plasma CXCL10, pg./mL^‡^ | All (100%) | 368.5 (176.9-712.8) |
| **Intrahepatic** |  |  |
| CXCL10 IHC, % surface coverage^\|\|^ | All (100%) | 0.4% (0.2-0.7) |
| Liver CXCL10 delta-delta Ct^‡^ | All (100%) | 15.3 (7.0-26.3) |
| Liver CXCR3 delta-delta Ct^‡^ | All (100%) | 0.99 (0.68-1.57) |
| Liver IFN-α delta-delta Ct^§^ | Not detected (0%) | not detected |
| Liver IFN-β, delta-delta Ct^§^ | 69% | 0.00 (0.0- 0.07) |
| Liver IFN-γ, delta-delta Ct^§^ | 95% | 6.77 (3.89-9.92) |
| LPS, IHC (% surface coverage)^¶^ | 100% | 0.86% (0.43-1.53%) |
| MPO, IHC (% surface coverage) | 100% | 0.62% (0.45-0.78%) |
|  |  |  |
| **Viral Measures** |  |  |
| Blood CD4+ T-cell CA-US HIV RNA, copies/10^6^ 18s^‡^ | 84% | 112 (14-381) |
| Blood CD4+ T-cell HIV DNA, copies/10^6^ cells | 100% | 1679 (768-4040) |
| Liver CA-US HIV RNA, copies/10^6^ 18s^¶^ | 61% | 0 (0-0.88) |
| Liver HIV DNA, copies/10^6^ cells^¶^ | 44% | 25 (0-108) |
| HBV rcDNA, copies/Geq^**^ | 100% | 9.29 (0.42-63.63) |
| HBV cccDNA, copies/Geq^**^ | 83% | 0.12 (0.00-0.37) |

All values are presented as median (25th-75th percentiles) unless otherwise stated;

n=39, except where specified: ^‡^n=37, ^§^n=35,  ^||^n=24, ^¶^=38, ^**^n=27.

HMGB1 high mobility group box-1, CCL-2 C-C motif chemokine 2, sCD14 soluble CD14, LPS lipopolysaccharide, IHC immunohistochemistry, CXCL10 C-X-C motif chemokine, pg. picograms, IHC immunohistochemistry, CXCR3 C-X-C motif chemokine receptor 3, IFN interferon, CA- cell associated, US unspliced, HIV human immunodeficiency virus, HBV hepatitis B virus, cccDNA covalently closed circular DNA, rcDNA relaxed circular DNA; Geq genome equivalent
