# Supplementary material for: Intrahepatic CXCL10 is strongly associated with liver fibrosis in HIV-Hepatitis B co-infection
Source: PLoS Pathog. 2020 Sep 8;16(9):e1008744. doi: 10.1371/journal.ppat.1008744 (PMC7521747; doi:10.1371/journal.ppat.1008744)
Supplement: S3 Table — Factors that had a statistically significant association with fibrosis are shaded in grey (indicating p<0.05). Darker shading has been used to indicate factors that were significantly associated with at least three liver enzymes. Partial eta squared values (η2) were obtained for the linear regression models to quantify the effect size, using Cohen’s benchmarks to categorise η2 as small (η2 = 0.01), medium (η2 = 0.06), and large (η2 = 0.14). n = 39, except where specified: ‡n = 37, §n = 35, ||n = 24, ¶n = 38, **n = 27 CI = Confidence Interval, ALP alkaline phosphatase, GGT γ-glutamyl transferase, ALT Alanine transaminase, AST Aspartate transaminase, CXCL10 C-X-C motif chemokine, pg. picograms, CXCR3 C-X-C motif chemokine receptor 3, IFN interferon, LPS lipopolysaccharide, HIV human immunodeficiency virus, HBV hepatitis B virus, CA- cell associated, cccDNA covalently closed circular DNA, rcDNA relaxed circular DNA, Geq genome equivalent. (DOCX) [file ppat.1008744.s008.docx]

#### S3 Table. Factors associated with liver enzymes by linear regression analysis on loge transformed outcome, or log10 transformed (plasma sCD14) adjusted for total CD4 count.

Factors that had a statistically significant association with fibrosis are shaded in grey (indicating p<0.05). Darker shading has been used to indicate factors that were significantly associated with at least three liver enzymes. Partial eta squared values (η2) were obtained for linear regression models to quantify the effect size, using Cohen’s benchmarks to categorise η2 as small (η2 = 0.01), medium (η2 = 0.06), and large (η2 = 0.14).

| **Variable** | | **ALP** | **GGT** | **AST** | **ALT** |
| --- | --- | --- | --- | --- | --- |
|  |  | **Linear Regression Coefficient (95%CI)**  **Partial Eta-Squared (η2), P-value** | | | |
| **Peripheral** | **Plasma CXCL10**^‡^ | 0.007 (-0.005, 0.020) 0.04, 0.247 | 0.027 (0.003, 0.051) 0.13, 0.028 | 0.008 (-0.001, 0.018) 0.08, 0.091 | 0.004 (-0.011, 0.019), 0.01, 0.633 |
|  | **CD4+ T cell CA-HIV RNA**^‡^ | 0.014 (0.009, 0.018) 0.50, <0.001 | 0.016 (0.003, 0.028) 0.016, 0.16 | 0.008 (0.003, 0.012) 0.24, 0.003 | 0.005 (-0.003, 0.013) 0.04, 0.221 |
|  | **Plasma HBV DNA log_10_** | -0.51 (-2.92, 1.91) 0.00, 0.671 | 2.77 (-2.12, 7.66) 0.04, 0.259 | 1.53 (-0.39, 3.45) 0.07, 0.115 | 2.05 (-0.84, 4.93) 0.05, 0.159 |
|  | **Plasma LPS**^‡.^ | 0.40 (0.15, 0.65) 0.24, 0.003 | 0.65 (0.12, 1.19) 0.15, 0.018 | 0.12 (-0.11, 0.34), 0.03, 0.308 | -0.04 (-0.39, 0.30) 0.00, 0.805 |
|  | **sCD14 log_10_** | 25.07 (-34.52, 84.66) 0.02, 0.399, | 85.35 (-34.98, 205.67) 0.05, 0.159 | 31.94 (-16.32, 80.19) 0.05, 0.188 | 4.80 (-68.88, 78.48) 0.00, 0.896 |
| **Liver** | **CXCL10 (Δ-Δ-Ct)**^‡^ | 0.26 (-0.24, 0.76) 0.03, 0.291 | 1.09 (0.12, 2.05), 0.13, 0.029 | 0.65 (0.31, 1.00), 0.30, 0.001 | 0.76 (0.20, 1.32) 0.18, 0.009 |
|  | **CXCR3 (Δ-Δ-Ct)**^‡^ | 1.43 (-7.01, 9.87) 0.00, 0.733 | 7.45 (-9.63, 24.52), 0.02, 0.382 | 4.56 (-2.14, 11.26), 0.05, 0.176 | 6.46 (-3.55, 16.47) 0.05, 0.199 |
|  | **IFN-γ (Δ-Δ-Ct)**^§^ | 0.35 (-0.92, 1.62) 0.01, 0.581 | 0.26 (-2.35, 2.87), 0.00, 0.841 | 0.16 (-0.88, 1.20) 0.00, 0.758 | -0.01 (-1.54, 1.52) 0.00 0.988, |
|  | **LPS (% area)^\|\|^** | 17.75 (-9.42, 44.93) 0.08, 0.189 | 65.31 (10.28, 120.34), 0.22, 0.022 | 8.76 (-12.06, 29.59), 0.04, 0.391 | -8.55 (-34.79, 17.68) 0.02, 0.505 |
|  | **HIV DNA**^¶^  **(copies/10^6^ cells)** | 0.02 (-0.02, 0.07) , 0.02, 0.360 | 0.09 (0.00, 0.18) 0.11, 0.041 | 0.02 (-0.02, 0.06) 0.03, 0.306 | -0.00 (-0.06, 0.06) 0.00, 0.993 |
|  | **HIV RNA**^¶^  **(copies/10^6^ cells)** | -0.40 (-2.48, 1.69) 0.00, 0.702 | 1.93 (-2.32, 6.18) 0.02, 0.362 | 1.24 (-0.43, 2.90) 0.06, 0.141 | 1.83 (-0.64, 4.30) 0.06, 0.142 |
|  | **HBV cccDNA****  **(copies/Geq)** | -3.13 (-12.38, 6.11) 0.02, 0.491 | 34.64, (20.31, 48.97) 0.51, <0.001 | 7.31 (-0.50, 15.12) 0.13, 0.065 | 1.29 (-10.10, 12.68) 0.04, 0.817 |
|  | **HBV rcDNA**** **(copies/Geq)** | -0.09 (-0.28, 0.10) 0.04, 0.331 | 0.27 (-0.14, 0.67), 0.07, 0.189 | 0.06 (-0.11, 0.23) 0.02, 0.474 | 0.02 (-0.21, 0.26) 0.00, 0.835 |

n=39, except where specified: ^‡^n=37, ^§^n=35, ^||^n=24, ^¶^n=38, **n=27

CI = Confidence Interval, ALP alkaline phosphatase, GGT γ-glutamyl transferase, ALT Alanine transaminase, AST Aspartate transaminase, CXCL10 C-X-C motif chemokine, pg. picograms, CXCR3 C-X-C motif chemokine receptor 3, IFN interferon, LPS lipopolysaccharide, HIV human immunodeficiency virus, HBV hepatitis B virus, CA- cell associated, cccDNA covalently closed circular DNA, rcDNA relaxed circular DNA, Geq genome equivalent.
