# Supplementary material for: Intrahepatic CXCL10 is strongly associated with liver fibrosis in HIV-Hepatitis B co-infection
Source: PLoS Pathog. 2020 Sep 8;16(9):e1008744. doi: 10.1371/journal.ppat.1008744 (PMC7521747; doi:10.1371/journal.ppat.1008744)
Supplement: S4 Table — Factors that had a statistically significant association with fibrosis are shaded in grey (indicating p<0.05). Partial eta squared values (η2) were obtained for the linear regression models to quantify the effect size, using Cohen’s benchmarks to categorise η2 as small (η2 = 0.01), medium (η2 = 0.06), and large (η2 = 0.14). n = 39, except: ‡n = 37, §n = 35, ||n = 24, ¶ n = 38, **n = 27, ††n = 33, ‡‡n = 22, §§n = 23, ||||n = 32, ¶¶n = 26; CI = Confidence Interval, CXCL10 C-X-C motif chemokine, CXCR3 C-X-C motif chemokine receptor 3, IFN interferon, LPS lipopolysaccharide, sCD14 soluble CD14, HIV human immunodeficiency virus, HBV hepatitis B virus, CA- cell associated, US unspliced; rcDNA relaxed circular DNA, Geq genome equivalent. (DOCX) [file ppat.1008744.s009.docx]

#### S4 Table. Factors associated with hepatic inflammatory markers, CXCL10, CXCR3, IFN-γ, LPS % area and circulating sCD14 by regression analysis on log_e_ transformed outcome, or log_10_ transformed (plasma sCD14) adjusted for total CD4 count.

Factors that had a statistically significant association are shaded in grey (indicating p<0.05). Partial eta squared values (η2) were obtained for the linear regression models to quantify the effect size, using Cohen’s benchmarks to categorise η2 as small (η2 = 0.01), medium (η2 =0.06), and large (η2 =0.14).

| **Variable** | | **Outcome**  **Linear Regression Coefficient (95%CI), Partial Eta-Squared (η2), p-value** | | | | |
| --- | --- | --- | --- | --- | --- | --- |
|  |  | Liver CXCL10 (Δ-Δ-Ct) | Liver CXCR3 (Δ-Δ-Ct) | Liver IFN-γ (Δ-Δ-Ct) | Liver LPS (% area) | Plasma sCD14 |
| **Peripheral** | HIV RNA log_10_ | 0.63 (0.11, 1.14) 0.15, 0.018^‡^ | 0.51 (0.16, 0.86) 0.20, 0.006^‡^ | 0.62 (0.21, 1.03) 0.23, 0.004^§^ | 0.51 (-0.02, 1.03) 0.16, 0.057^\|\|^ | 0.11 (0.04, 0.17) 0.25, 0.001 |
|  | CD4+ CA-US HIV RNA | 0.00 (-0.00, 0.00) 0.06, 0.172^§^ | 0.00 (-0.00, 0.00) 0.01, 0.494^§^ | -0.00 (-0.00, 0.00) 0.00, 0.977^††^ | 0.00 (-0.00,0.00) 0.04, 0.376^‡‡^ | 0.00 (-0.00, 0.00) 0.07, 0.130^‡^ |
|  | CD4+ T cell HIV DNA | 0.00 (-0.00, 0.00) 0.04, 0.245^‡^ | 0.00 (-0.00, 0.00) 0.04, 0.228^‡^ | 0.00 (-0.00, 0.00) 0.04, 0.251^§^ | 0.00 (-0.00, 0.00) 0.11, 0.126^\|\|^ | 0.00 (0.00, 0.00) 0.22, 0.003 |
|  | HBV DNA log_10_ | 0.13 (0.03, 0.22) 0.19, 0.009^‡^ | 0.00 (-0.07, 0.07) 0.00, 0.997^‡^ | -0.05 (-0.13, 0.04) 0.04, 0.246^§^ | 0.01 (-0.12, 0.14) 0.00, 0.833^\|\|^ | 0.01 (-0.00, 0.02) 0.08, 0.076 |
|  | Plasma sCD14 log_10_ | 1.21 (-1.40, 3.82) 0.03, 0.353^‡^ | -0.11 (-1.96, 1.74) 0.00, 0.904^‡^ | 0.08 (-2.14, 2.30) 0.00, 0.943^§^ | 3.22 (0.44, 6.00) 0.22, 0.025^\|\|^ |  |
|  | Plasma CXCL10 | 0.0003 (-0.0002, 0.0008) 0.03, 0.300^‡^ | 0.0004 (0.0001, 0.0007) 0.16, 0.015^‡^ | 0.0004 (-0.0000, 0.0008) 0.10, 0.066^§^ | 0.0006 (0.0002, 0.0011) 0.31, 0.009^‡‡^ | 0.0000 (-0.0000, 0.0001) 0.04, 0.234^‡^ |
| **Liver** | Liver LPS  (% area) | 0.46 (-0.48, 1.40) 0.05, 0.318^‡‡^ | 0.44 (-0.27, 1.15) 0.08, 0.212^‡‡^ | 0.84 (-0.03, 1.70) 0.18, 0.058^‡‡^ |  | 0.14 (0.01, 0.27) 0.18, 0.042^\|\|^ |
|  | Liver CA-US RNA | 0.07 (-0.01, 0.16) 0.09, 0.073^‡^ | 0.07 (0.02, 0.13) 0.18, 0.010^‡^ | 0.04 (-0.03, 0.11) 0.04, 0.240^§^ | 0.19 (-0.00, 0.38) 0.18, 0.051^§§^ | -0.00 (-0.01, 0.01) 0.01, 0.653^¶^ |
|  | Liver HIV DNA | 0.002 (0.000, 0.004) 0.15, 0.019^‡^ | 0.002 (0.001, 0.003) 0.36, <0.001^‡^ | 0.002 (0.001, 0.004) 0.24, 0.003^§^ | 0.002 (0.001, 0.004) 0.32, 0.006^§§^ | 0.000 (-0.000, 0.000) 0.08, 0.100^¶^ |
|  | HBV rcDNA  (copies/Geq) | 0.006 (-0.001, 0.013) 0.11, 0.110^\|\|\|\|^ | -0.002 (-0.007, 0.002) 0.05, 0.285^\|\|\|\|^ | -0.007 (-0.013, -0.001) 0.21, 0.029^\|\|^ | -0.002 (-0.011, 0.008) 0.01, 0.737^¶¶^ | 0.000 (-0.001, 0.001) 0.00, 0.825** |

n=39, except where specified, ^‡^n=37, ^§^n=35, ^||^n=24, ^¶^n=38, **n=27, ^††^n=33, ^‡‡^n=22, ^§§^n=23, ^||||^n=26, ^¶¶^n =18

CI = Confidence Interval, CXCL10 C-X-C motif chemokine, CXCR3 C-X-C motif chemokine receptor 3, IFN interferon, LPS lipopolysaccharide, sCD14 soluble CD14, HIV human immunodeficiency virus, HBV hepatitis B virus, CA- cell associated, US unspliced; rcDNA relaxed circular DNA, Geq genome equivalent.
